# SCOOP10 and SCOOP12 peptides act through MIK2 receptor-like kinase to antagonistically regulate *Arabidopsis* leaf senescence

**DOI:** 10.1016/j.molp.2024.10.010

**Published:** 2024-12-02

**Authors:** Zhenbiao Zhang, Nora Gigli-Bisceglia, Wei Li, Saijie Li, Jie Wang, Junfeng Liu, Christa Testerink, Yongfeng Guo

**Affiliations:** 1 Tobacco Research Institute, Chinese Academy of Agricultural Sciences, Qingdao, China; 2 Graduate School of Chinese Academy of Agricultural Sciences, Beijing, China; 3 Laboratory of Plant Physiology, Wageningen University & Research, Wageningen, the Netherlands; 4 Plant Stress Resilience, Institute of Environmental Biology, Utrecht University, Utrecht, the Netherlands; 5 State Key Laboratory of Maize Bio-breeding, Ministry of Agriculture Key Laboratory for Crop Pest Monitoring and Green Control, Joint International Research Laboratory of Crop Molecular Breeding, China Agricultural University, Beijing, China

**Keywords:** leaf senescence, MIK2, SCOOP10, SCOOP12, small signaling peptides, receptor-like kinase

## Abstract

Leaf senescence plays a critical role in a plant’s overall reproductive success due to its involvement in nutrient remobilization and allocation. However, our current understanding of the molecular mechanisms controlling leaf senescence remains limited. In this study, we show that the receptor-like kinase MALE DISCOVERER 1-INTERACTING RECEPTOR-LIKE KINASE 2 (MIK2) functions as a negative regulator of leaf senescence. We found that the SERINE-RICH ENDOGENOUS PEPTIDE 12, previously known to physically interact with MIK2, competes with SCOOP10 to regulate MIK2-dependent leaf senescence. We observed that increased expression of SCOOP10 or the application of exogenous SCOOP10 peptides accelerated leaf senescence in a MIK2-dependent manner. Conversely, SCOOP12 acted as a suppressor of MIK2-dependent leaf senescence regulation. Biochemical assays showed that SCOOP12 enhances while SCOOP10 diminishes MIK2 phosphorylation. Thus, the SCOOP12-MIK2 module might function antagonistically on SCOOP10-MIK2 signaling at late senescing stages, allowing for fine-tuned modulation of the leaf senescence process. Our study sheds light on the complex mechanisms underlying leaf senescence and provides valuable insights into the interplay between receptors, peptides, and the regulation of plant senescence.

## Teaser

This study reveals that two peptides from the SERINE-RICH ENDOGENOUS PEPTIDE family, specifically SCOOP10 and SCOOP12, bind to the Receptor-Like Kinase (RLK) MIK2 and antagonistically regulate the progression of leaf senescence in *Arabidopsis*. The results highlight a complex and tightly controlled mechanism that governs leaf aging.

## Introduction

Senescence, a crucial biological event in plant development, involves the coordinated breakdown of cellular components to recycle nutrients, aiding adaptation to changing environments. It optimizes nutrient storage and reuse, particularly in leaves, the primary photosynthetic organs. Initiation of leaf senescence prompts various physiological changes, including chlorophyll (Chl) reduction and macromolecule degradation, enabling nutrient recycling that supports the growth of seeds and fruits (Yang et al., 2014; Kim et al., 2018; Woo et al., 2019; Guo et al., 2021). Efficient senescence is vital for non-perennial plants like wheat and sorghum, as it maximizes nutrient use and prevents yield loss during adverse conditions (Tollenaar and Daynard, 1978; Naruoka et al., 2012). Understanding the molecular mechanisms behind leaf senescence is critical for improving agricultural productivity, especially in challenging climates. Senescence is a programmed cell death process, which is triggered by endogenous cues and environmental factors, with phytohormones like ethylene, cytokinins, abscisic acid, jasmonic acid, and salicylic acid playing significant roles (Guo and Gan, 2014). Recently, peptide hormones have emerged as signaling molecules controlling several aspects of plant developmental processes, including senescence (Hirakawa and Sawa, 2019; Han et al., 2022). These plant endogenous peptides, typically consisting of fewer than 100 amino acids, are synthesized within the cytosol and subsequently secreted into the apoplast, where they are thought to interact with receptor protein kinases located at the plasma membrane (Tavormina et al., 2015; Gust et al., 2017; Hou et al., 2021a). For example, the CLAVATA3/EMBRYO-SURROUNDING REGION-RELATED (CLE) peptide family members CLE14 and CLE42 negatively regulate age-dependent leaf senescence (Han et al., 2022). In fact, loss-of-function mutations in *CLE14* or *CLE42* caused an accelerated senescence, while the overexpression of *CLE14* or *CLE42* led to a delayed leaf senescence (Zhang et al., 2022a, 2022b). Additionally, the INFLORESCENCE DEFICIENT IN ABSCISSION-LIKE6 (IDL6) peptide has been reported to positively modulate leaf senescence in *Arabidopsis thaliana*. The *idl6* mutants display delayed leaf senescence, while overexpression of *IDL6* accelerates leaf senescence (Guo et al., 2022). Furthermore, the plant elicitor peptide PEP1, one of the first plant phytocytokines identified to regulate plant immune responses (Huffaker et al., 2006; Bartels et al., 2013; Hou et al., 2021a), has also been shown to function as a leaf senescence modulator in *Arabidopsis* (Gully et al., 2015). SERINE-RICH ENDOGENOUS PEPTIDES (SCOOPs) are an emerging class of phytocytokines present specifically in *Brassicaceae*, and have been recently reported to be implicated in controlling plant immunity and development (Gully et al., 2019; Hou et al., 2021b; Rhodes et al., 2021). The precursors of SCOOPs, PROSCOOPs (Gully et al., 2019) must undergo active N terminus proteolysis to generate C terminus active SCOOP peptides. In 2021, 23 SCOOPs (SCOOP1–23) were identified in *Arabidopsis* (Hou et al., 2021b), but only recently has the family been suggested to contain at least 50 members (Yang et al., 2023). Among them, SCOOP12 has been shown to bind to the Leucine-Rich Repeat (LRR) Receptor-Like Kinase (RLK) MALE DISCOVERER 1-INTERACTING RECEPTOR-LIKE KINASE 2 (MIK2) to activate the plant immune response to *Fusarium oxysporum* (Hou et al., 2021b; Rhodes et al., 2021). PROSCOOP10 was recently found to be processed at two encoded regions to produce two distinct forms of SCOOP peptides (SCOOP10#1 and SCOOP10#2) (Guillou et al., 2022). SCOOP10#2 (referred to as SCOOP10 in this article) has also been shown to have dual effects: inducing plant immune responses and inhibiting root elongation (Hou et al., 2021b; Rhodes et al., 2021). MIK2 has been reported to recognize the *Fusarium*-derived SCOOP-like motifs that are essential to trigger the MIK2-dependent immunity. However, MIK2 has also been suggested to be the receptor of the *Arabidopsis* SCOOP family members, including SCOOP4, 6, 8, 10, 12, 13, 14, 15, 20, and 23 (Hou et al., 2021b; Rhodes et al., 2021). In this study, we report a novel role for MIK2 and SCOOP10/12 peptides in regulating leaf senescence. We show that MIK2 plays a negative regulatory role in both age-dependent and dark-induced leaf senescence. Mutants with disrupted function of *MIK2* exhibit precocious senescence, whereas transgenic plants expressing *MIK2* under a senescence-induced promoter show delayed senescence phenotypes. Plants lacking *PROSCOOP10* display a delayed senescence, while exogenous application of synthetic SCOOP10 peptides in detached leaves accelerates leaf senescence in a MIK2-dependent manner. By contrast, loss-of-function *scoop12* mutants exhibit accelerated leaf senescence phenotypes and SCOOP12 peptides inhibit senescence in a MIK2-dependent manner. Both SCOOP10 and SCOOP12 peptides bind to the extracellular domain of MIK2, indicating that MIK2 serves as a receptor for both senescence-promoting (SCOOP10) and senescence-inhibiting (SCOOP12) peptides. Biochemical assays further revealed that SCOOP10 negatively regulates but SCOOP12 enhances MIK2 kinase phosphorylation. Notably, SCOOP12 can outcompete SCOOP10 in their shared receptor’s function, as SCOOP12 triggers MIK2 phosphorylation even in the presence of SCOOP10. Our results suggest that SCOOP10-MIK2 complex formation is antagonistically regulated by SCOOP12, implicating that the SCOOP10-MIK2 and SCOOP12-MIK2 modules might operate antagonistically to fine-tune senescence in *Arabidopsis* leaves.

## Results

### MIK2 negatively regulates leaf senescence

**Figure 1:**
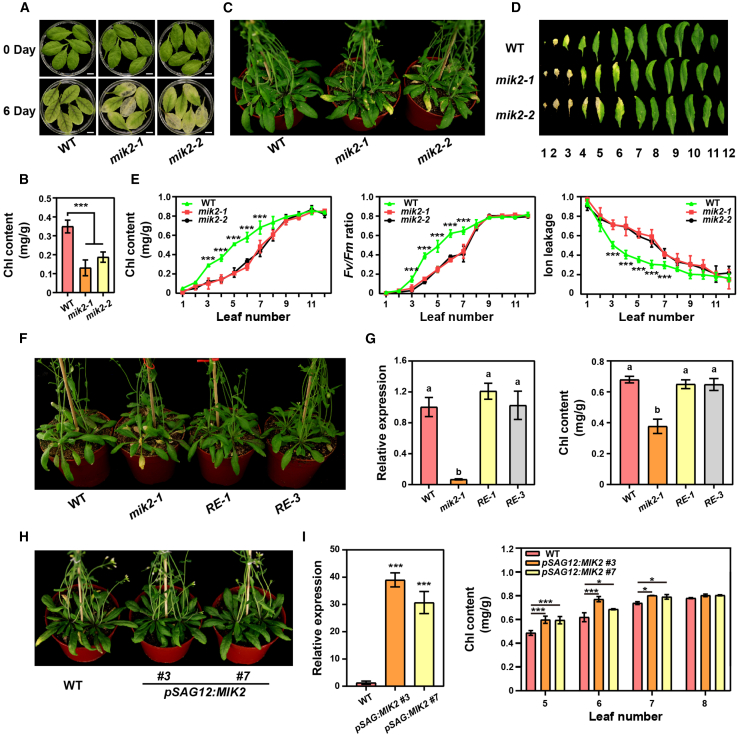
MIK2 negatively regulates both age-dependent and dark-induced leaf senescence. **(A)** Leaf senescence phenotype was assessed in detached sixth rosette leaves obtained from 4-week-old *mik2-1* and *mik2-2* mutants and Col-0 (WT) plants after 6 days of dark treatment. Representative results from three independent experiments are presented. **(B)** Chlorophyll (Chl) content was analyzed in the sixth detached leaves as indicated in **(A)**. Bars represent means ± SDs of three biological replicates (*n* = 6 leaves). Asterisks denote statistically significant differences compared to WT according to one-way ANOVA (α = 0.05), followed by Bonferroni correction for multiple comparisons (∗*p* ≤ 0.05; ∗∗*p* ≤ 0.01; ∗∗∗*p* ≤ 0.001). **(C)** Adult plant phenotype of *mik2* mutants and WT plants was observed after 40 days of growth under long-day conditions. **(D)** Senescence progression was evaluated in the first 12 leaves isolated from the plants depicted in **(C)**. **(E)** Measurements of Chl content, PSII efficiency expressed as Fv/Fm ratio, and ion leakage were performed in adult plants of WT, *mik2-1*, and *mik2-2* mutants. Bars represent means ± SDs of three biological replicates (*n* = 3 leaves). Asterisks indicate significant differences compared to WT (one-way ANOVA [α = 0.05], Bonferroni post hoc test; ∗*p* ≤ 0.05; ∗∗*p* ≤ 0.01; ∗∗∗*p* ≤ 0.001). **(F)** Leaf senescence phenotype was examined in two independent homozygous transgenic lines expressing *pMIK2*:*MIK2* (*RE-1* and *RE-3*) in *mik2-1* mutant background. **(G)** Analysis of *MIK2* expression and measurement of Chl content were conducted in the sixth rosette leaf of plants in **(F)**. Bars represent means ± SDs of three biological replicates (*n* = 3 leaves). Statistical analysis was performed using one-way ANOVA (α = 0.05). Groups marked with different letters show statistically significant differences between group means (Bonferroni post hoc test). **(H)** Phenotypical analysis conducted on *pSAG12*:*MIK2*-expressing plants grown for 45 days. Two independent lines, *pSAG12*:*MIK2* #3 and *pSAG12*:*MIK2* #7, were tested. Three biological replicates were included in each experiment. **(I)** Relative expression of *MIK2* and Chl content were analyzed using the fifth, sixth, seventh, and eighth rosette leaves of the plants described in **(H)**. Bars represent means ± SDs of three biological replicates (*n* = 3 leaves). Asterisks indicate significant differences compared to WT (one-way ANOVA, Bonferroni post hoc test; ∗*p* ≤ 0.05; ∗∗*p* ≤ 0.01; ∗∗∗*p* ≤ 0.001).

To identify new receptor-like kinases (RLKs) regulating leaf senescence, we conducted a phenotypic analysis using a collection of over 200 T-DNA mutants obtained from the Arabidopsis Biological Resource Center (ABRC). These mutants had mutations in RLK genes (Supplemental Table 1). Here, we report that plants lacking *MIK2* showed accelerated dark- and age-induced leaf senescence. Two independent T-DNA insertion lines, *mik2-1* (SALK_061769) and *mik2-2* (CS419958) (Supplemental Figure 1A), were used for loss-of-function analysis. When compared to the corresponding wild-type (WT) plants (Col-0), detached leaves from the *mik2* mutants exhibited accelerated senescence after 6 days of darkness (Figures 1A and 1B). Lack of *MIK2* also caused premature leaf senescence in plants grown under controlled laboratory conditions (Figures 1C and 1D). Consistently, rosette leaves of *mik2* plants exhibited lower Chl content, lower photochemical efficiency of photosystem II (PSII) (Fv/Fm ratio) and higher membrane ion leakage than that of WT (Figure 1E). We found that *MIK2* is mainly expressed in aging leaves (Supplemental Figures 1B and 1C) and that MIK2 proteins were confirmed to be localized on the plasma membrane (Supplemental Figure 1D). The premature senescence phenotype observed in the *mik2* mutants was reversed to the WT phenotype by introducing the *MIK2* coding region under the control of a 2200-bp *MIK2* native promoter (Figures 1F and 1G). Two individual transgenic lines with senescence-induced expression of *MIK2* driven by *pSAG12* (senescence-specific promoter) (Lohman et al., 1994; Gan and Amasino, 1995, 1997) were generated and displayed delayed leaf senescence with increased Chl content at the fifth, sixth, and seventh rosette leaves in comparison to WT plants (Figures 1H and 1I), highlighting that the presence of MIK2 was sufficient to inhibit leaf senescence, supporting the role of MIK2 in the regulation of leaf senescence.

### SCOOP10 positively regulates leaf senescence

**Figure 2:**
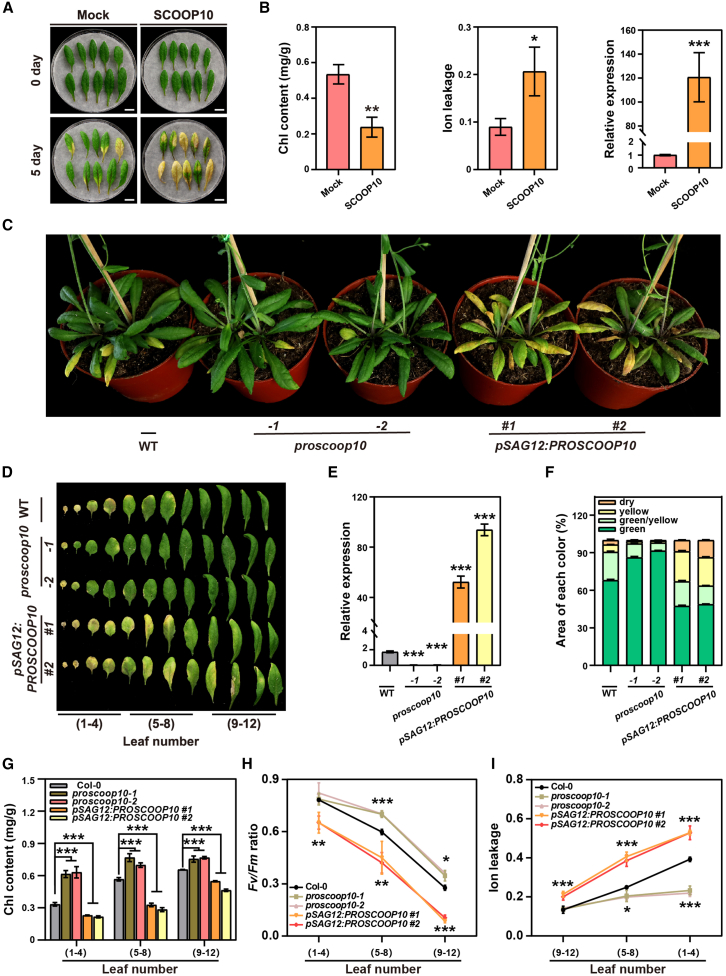
SCOOP10 positively regulates leaf senescence. **(A)** Leaf senescence was analyzed in the fifth and sixth rosette leaves derived from 4-week-old WT plants treated with chemically synthesized SCOOP10 peptides (1 μM) for 5 days. Representative results are shown from three independent experiments. **(B)** Chl content, ion leakage, and *SAG12* expression were analyzed in the detached leaves shown in **(A)**. Bars represent means ± SDs of three biological replicates (*n* = 10 leaves). Asterisks denote statistically significant differences compared to WT, as determined by Student’s t test (∗*p* ≤ 0.05; ∗∗*p* ≤ 0.01; ∗∗∗*p* ≤ 0.001). **(C** **and D)** Rosette leaves of *proscoop10* mutants *(proscoop10-1* and *proscoop10-2), pSAG12*:*PROSCOOP10* (1 and 2), and corresponding WT plants were isolated and grouped into leaf numbers 1–4, 5–8, and 9–12. **(E)** *PROSCOOP10* expression in *proscoop10* mutants, *pSAG12*:*PROSCOOP10* #1 and #2, and corresponding WT. Bars represent means ± SDs of three biological replicates (*n* = 4 leaves). Asterisks indicate significant differences compared to WT (one-way ANOVA with Bonferroni correction; ∗*p* ≤ 0.05; ∗∗*p* ≤ 0.01; ∗∗∗*p* ≤ 0.001). **(F)** Area of each color of *proscoop10* mutants and the *pSAG12*:*PROSCOOP10* #1 and #2 transgenic lines grown under long-day conditions for 45 days. **(G–I)** Chl content **(G)**, Fv/Fm ratio **(H)**, and ion leakage **(I)** were measured in the detached leaves of plants presented in **(C)** and **(D)**. Bars represent means ± SDs of three biological replicates (*n* = 4 leaves). Asterisks indicate statistically significant differences compared to WT (one-way ANOVA with Bonferroni correction; ∗*p* ≤ 0.05; ∗∗*p* ≤ 0.01; ∗∗∗*p* ≤ 0.001).

**Figure 3:**
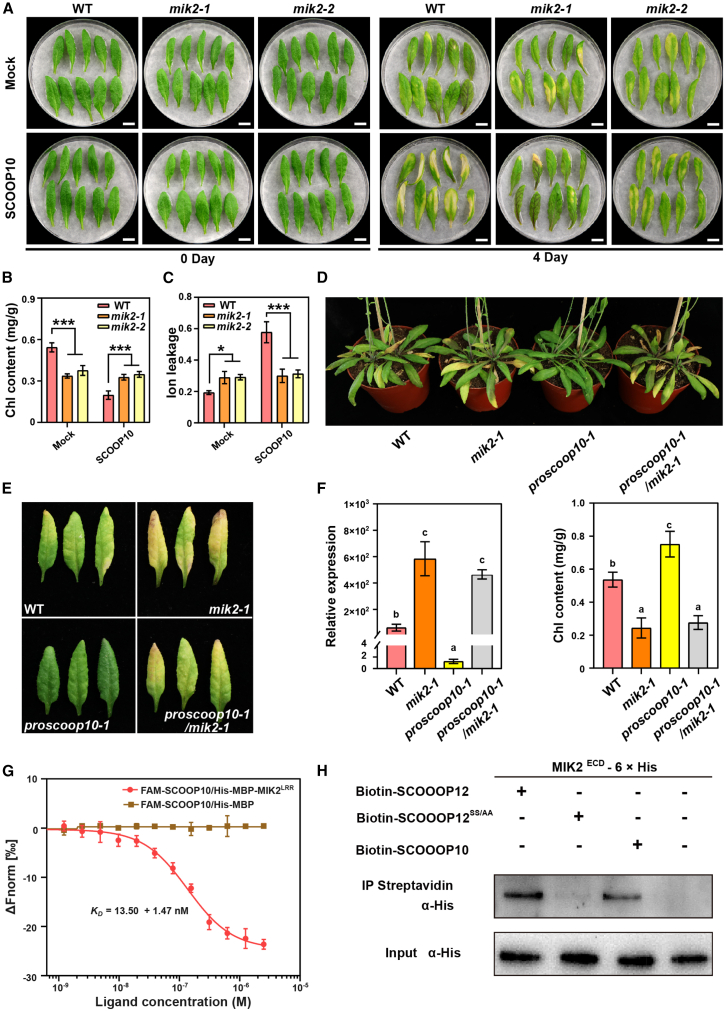
SCOOP10 regulates leaf senescence in a MIK2-dependent manner. **(A)** Leaf senescence was analyzed in the fifth and sixth rosette leaves derived from 4-week-old *mik2* mutants and corresponding WT plants upon treatments with synthesized SCOOP10 peptides (1 μM) for 2 and 4 days under light conditions. **(B and C)** Changes in Chl content **(B)** and **(C)** ion leakage of detached leaves shown in **(A)** after 4 days of treatments. Data are presented as means ± SDs from three independent experiments (*n* = 10 leaves per replicate). Asterisks denote statistically significant differences (one-way ANOVA followed by Bonferroni correction; ∗*p* ≤ 0.05; ∗∗∗*p* ≤ 0.001). **(D)** Leaf senescence phenotype of 45-day-old *proscoop10-1 mik2-1* double mutants, *proscoop10-1* and *mik2-1* single mutants, and corresponding WT plants grown under long-day conditions. **(E)** Senescence phenotype of the sixth rosette leaves isolated from the plants in **(D)**. **(F)** Chl content and transcript levels of *SAG12* measured in the sixth rosette leaves from the plants presented in **(D)**. Bars represent means ± SDs of three independent experiments, each containing six leaves. Statistical analysis was performed using one-way ANOVA (α = 0.05). Groups marked with different letters show statistically significant differences between group means (Bonferroni post hoc test). **(G)** Determination of the binding affinity between SCOOP10 peptides with a fluorescent FAM label on the N terminus and the ectodomain of MIK2 using microscale thermophoresis (MST). FAM-SCOOP10/MBP was used as a negative control in the binding assay. The experiments were repeated three times with similar results. **(H)** A pull-down assay was conducted to investigate the interaction between the ectodomains of MIK2, tagged with 6× His, and biotinylated peptides SCOOP10, SCOOP12, and SCOOP12SS/AA (negative control). The MIK2 ectodomains, expressed in insect cells, were incubated in a binding buffer with the biotinylated peptides (SCOOP10, SCOOP12, and SCOOP12SS/AA) for 30 min. Streptavidin magnetic beads were used, and both the bound and input proteins were then analyzed via Western blot using an anti-His antibody to detect the presence of MIK2. The experiments were repeated three times with similar results.

Because MIK2 was recently reported to function as the receptor of the phytocytokine SCOOPs (Hou et al., 2021b; Rhodes et al., 2021), we tested whether 23 of the 50 identified SCOOP peptides played a regulatory role in leaf senescence. *PROSCOOP* expression was analyzed during different leaf developmental stages (Supplemental Figure 2). Several *PROSCOOP*s showed a significant change in expression during leaf senescence. For instance, *PROSCOOP10* expression was found upregulated at the early stages of senescence, while *PROSCOOP12* was strongly upregulated in late senescent leaves (Supplemental Figure 2). Moreover, the exogenous application of several synthesized SCOOP peptides caused changes in senescence progression in detached leaves (Supplemental Figures 3A and 3B). Application of SCOOP10#2 but not SCOOP10#1 peptides led to a premature senescence phenotype in detached leaves (Figures 2A and 2B and Supplemental Figures 3A and 3B). Exogenous application of the SCOOP10#2 (hereafter, SCOOP10) peptide on intact rosette leaves caused significantly accelerated leaf senescence, which was associated with reduced Chl content and Fv/Fm ratio (Supplemental Figures 4A and 4B). To better understand the role of SCOOP10 in leaf senescence, two loss-of-function *proscoop10* T-DNA mutant lines (*proscoop10-1*, SALK_080439; and *proscoop10-2*, SALK_027949) were analyzed. Both lines displayed a delayed leaf senescence phenotype (Figures 2C and 2D) with higher Chl content, Fv/Fm ratio, and lower ion leakage (Figures 2G–2I). Transgenic *pSAG12:PROSCOOP10* lines in Col-0 (WT) showed an earlier senescence phenotype compared to the non-transformed WT plants (Figures 2C–2E). *pSAG12:PROSCOOP10* plants were in fact characterized by increased *PROSCOOP10* expression (Figure 2E), yellower leaves (Figure 2F), decreased Chl content, Fv/Fm ratio, and increased ion leakage rate (Figures 2G–2I). Detached leaves from *proscoop10* mutants displayed a delayed senescence phenotype after 8 days of dark treatments (Supplemental Figures 4C and 4D). To investigate whether MIK2 is required for PROSCOOP10-triggered leaf senescence, detached leaves of *mik2* mutants were treated with SCOOP10 synthetic peptide (Figures 3A–3C). *mik2* mutant lines were nonresponsive to SCOOP10 peptide application, strongly suggesting that MIK2 is required for the perception of SCOOP10 and for the SCOOP10-mediated induction of leaf senescence. To confirm this, we generated a *proscoop10-1 mik2-1* double mutant line. The *proscoop10-1 mik2-1* plants were characterized by premature senescence phenotypes when compared to *proscoop10-1* or WT plants, likely reflecting the *mik2-1* mutant phenotype (Figure 3D and 3E). Consistent with the premature senescence phenotype, the *proscoop10-1 mik2-1* double mutant displayed lower Chl content and *SAG12* expression when compared to that of the controls (Figure 3F). Furthermore, a microscale thermophoresis (MST) assay was performed to determine the ability of MIK2 ectodomain (expressed in *Escherichia coli* cells) in binding the SCOOP10 peptide labeled with FAM at the N-terminus (5′-FAM-SCOOP10) (Figure 3G). The biological functions of the 5′-FAM-SCOOP10 peptide (i.e., induce leaf senescence and/or root growth inhibition) (Hou et al., 2021b; Rhodes et al., 2021) were confirmed to be similar to those triggered by the unlabeled peptide (Supplemental Figures 5A and 5B). The results of the MST analysis indicated that the FAM-SCOOP10 peptide was able to bind to the ectodomain of MIK2 with a dissociation constant (K_D_) of 13.50 ± 1.47 nM (Figure 3G), which suggested a direct interaction between SCOOP10 and MIK2 *in vitro*. To validate our observations, we performed a pull-down assay where we used the purified His-tagged MIK2 ectodomain and combined it either with biotinylated SCOOP10 or SCOOP12 (Figure 3H). As a negative control, we used SCOOP12^SS/AA^, previously described as being unable to bind the MIK2 ectodomain (Hou et al., 2021b). Taken together, the results show that SCOOP10 interacts with MIK2 and that PROSCOOP10 mediates leaf senescence in a MIK2-dependent manner. These findings collectively indicate that SCOOP10 plays a positive regulatory role in both age-dependent and dark-induced leaf senescence.

### SCOOP12 negatively regulates leaf senescence in a MIK2-dependent manner

**Figure 4:**
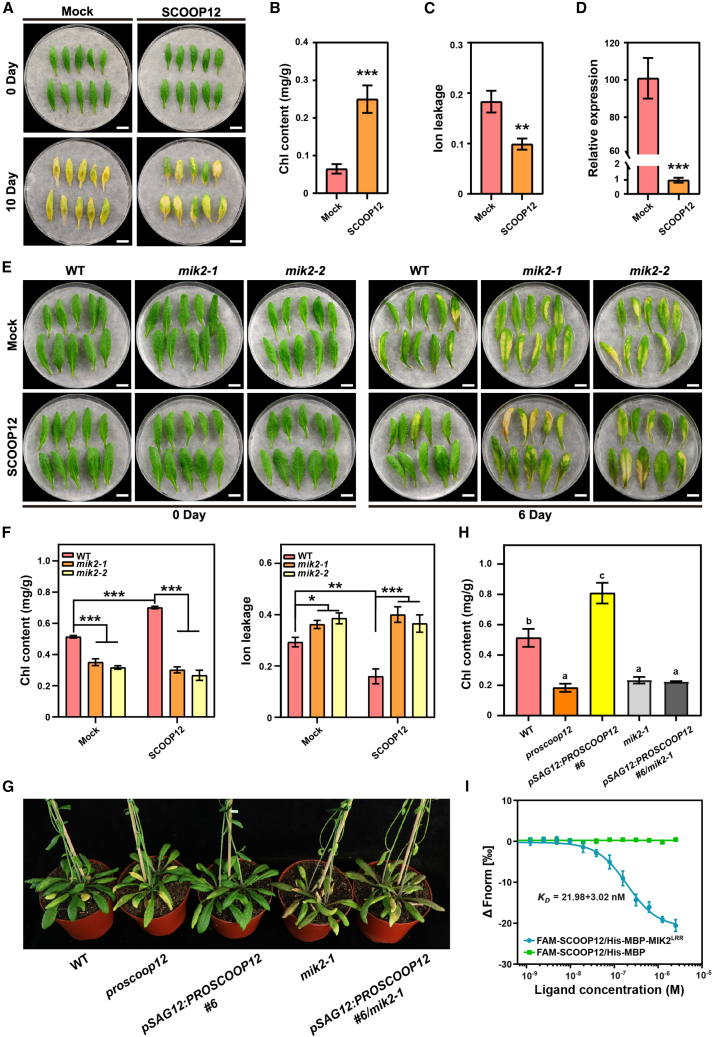
SCOOP12 negatively regulates leaf senescence in an MIK2-dependent manner. **(A)** Leaf senescence phenotype was analyzed in the fifth rosette leaves from 4-week-old plants upon treatments with synthesized SCOOP12 peptides (1 μM) for 10 days. **(B–D)** Chl content **(B)**, ion leakage **(C),** and *SAG12* expression **(D)** were measured in detached leaves shown in **(A)** 10 days after treatments. Bars represent means ± SDs from three independent experiments (*n* = 10 leaves per replicate). Asterisks indicate statistically significant differences compared to WT, determined by Student’s t test (∗∗*p* ≤ 0.01; ∗∗∗*p* ≤ 0.001). **(E)** Senescence phenotypes analyzed in the fifth and sixth rosette leaves from 4-week-old *mik2* mutants and corresponding WT treated with synthesized SCOOP12 peptides (1 μM) for 6 days under light condition. **(F)** Chl content and ion leakage were measured in detached leaves shown in **(E)**. Bars represent means ± SDs of three independent experiments, with 10 leaves per replicate. Asterisks denote statistically significant differences between treatments and genotypes, determined by two-way ANOVA, followed by Bonferroni correction (∗*p* ≤ 0.05; ∗∗*p* ≤ 0.01; ∗∗∗*p* ≤ 0.001). **(G)** Senescence phenotype was analyzed in 6-week-old *pSAG12*:*SCOOP12* #6 plants in the *mik2-1* background, *pSAG12*:*SCOOP12* #6, *proscoop12*, *mik2-1*, and WT plants grown under long-day conditions. **(H)** Chl content of the sixth rosette leaves collected from plants shown in **(G)**. Bars represent means ± SDs of three independent experiments. Statistical analysis was performed using one-way ANOVA (α = 0.05). Groups marked with different letters show statistically significant differences between group means (Bonferroni post hoc test). **(I)** MST analysis of binding affinity between SCOOP12 peptides and the ectodomain of MIK2. The experiments were repeated three times with similar results.

Because SCOOP12 peptide was recently reported to directly bind the ectodomain of MIK2 *in vitro* (Hou et al., 2021b), we also studied the function of SCOOP12 in regulating leaf senescence. Exogenous application of SCOOP12 peptides significantly inhibited senescence (Figures 4A–4D), suggesting that SCOOP12 might function as a negative regulator of senescence. Further analyses showed that detached leaves of *mik2* mutants were nonresponsive to the exogenous application of SCOOP12 peptide (Figures 4E and 4F), indicating that SCOOP12-mediated inhibition of leaf senescence requires a functional MIK2. Furthermore, loss-of-function *proscoop12* mutant lines (Stahl et al., 2022) displayed premature senescence, while senescence-driven expression of *PROSCOOP12* (*pSAG12:PROSCOOP12* transgenic plants) led to delay in leaf senescence (Figures 4G and 4H). To further investigate the genetic interaction between PROSCOOP12 and MIK2, we found that double mutants (*pSAG12:PROSCOOP12 #6 mik2-1* double mutants) displayed an earlier senescence phenotype comparable to that of the *mik2* loss-of-function mutants (Figures 4G and 4H). Consistent with the previous findings (Hou et al., 2021b), the MST results indicated a direct binding between the ectodomains of MIK2 and SCOOP12 with a K_D_ of 21.98 ± 3.02 nM (Figure 4I). These data suggest that the senescence-inhibiting effect of SCOOP12 is genetically dependent on MIK2. These findings strongly support the hypothesis that not only SCOOP10 but also SCOOP12 interacts with MIK2 to modulate senescence progression.

### SCOOP10 and SCOOP12 act antagonistically in regulating leaf senescence through MIK2

**Figure 5:**
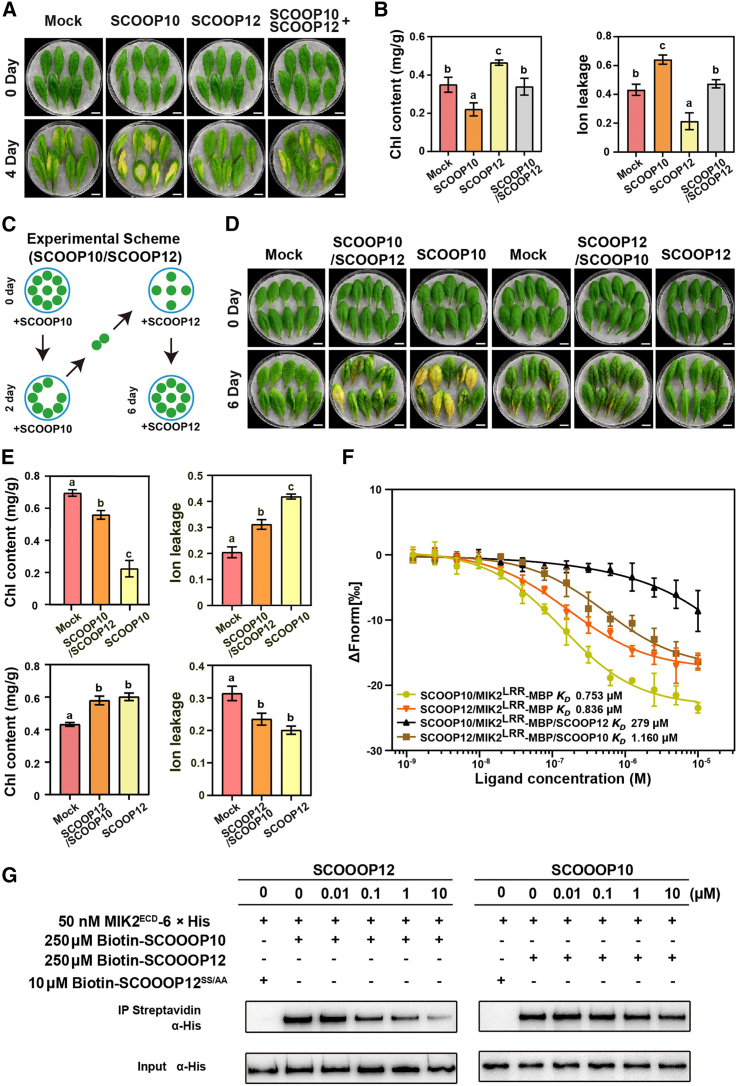
SCOOP12 competes with SCOOP10 for MIK2 interaction in regulating leaf senescence. **(A)** Senescence phenotype of the sixth rosette leaves from 30-day-old WT plants was analyzed upon treatments with 1 μM SCOOP10, SCOOP12, SCOOP10 plus SCOOP12 (SCOOP10/SCOOP12) peptides, or mock (ddH_2_O) after 4 days under long-day conditions. **(B)** Chl content and ion leakage were measured in detached leaves as shown in **(A)**. Bars represent means ± SDs from three biological replicates (*n* = 8 leaves per replicate). Different letters indicate statistically significant differences between treatments, as determined by one-way ANOVA (α = 0.05) and Bonferroni post hoc test. **(C)** The experimental scheme for competitive assay between SCOOP12 and SCOOP10 peptides in regulating leaf senescence. The fifth and sixth rosette leaves from 4-week-old plants were first pretreated by SCOOP12 peptides for 2 days, then transferred to ddH_2_O containing SCOOP10 peptides and incubated for 4 additional days under long-day conditions. **(D)** Senescence phenotype analyzed in detached leaves pretreated either with SCOOP10 or SCOOP12 peptides for 2 days and then treated in SCOOP12 or SCOOP10 peptides for 4 additional days. **(E)** Chl content and ion leakage measured in detached leaves shown in **(D)**. Bars show means ± SDs from three biological replicates (*n* = 8 leaves per replicate). Different letters indicate statistically significant differences, determined by one-way ANOVA (α = 0.05) followed by Bonferroni post hoc test. **(F)** Competitive inhibition analysis of SCOOP12 and FAM-SCOOP10 peptides in binding the MIK2 ectodomain was performed with MST. **(G)** Pull-down assays demonstrating competitive inhibition between varying concentrations of SCOOP12 or SCOOP10 peptides for binding to the 6× His-tagged ectodomains of MIK2. A 50-nM MIK2 ectodomain was incubated with 250 nM biotinylated SCOOP10 or SCOOP12 peptides in binding buffer for 1 h, followed by the addition of specified concentrations of SCOOP10/12 peptides. Western blotting was used to detect the bound and input proteins with an anti-His antibody.

To investigate the interactions between SCOOP10 and SCOOP12 in the regulation of the MIK2-dependent leaf senescence, WT detached leaves were treated with SCOOP10 and SCOOP12 peptides simultaneously. Interestingly, SCOOP10-triggered leaf senescence was alleviated by SCOOP12 peptide application (Figures 5A and 5B), suggesting an antagonistic effect between the two peptides. To explore this antagonistic mechanism further, a sequential treatment assay was designed, where detached leaves were initially pretreated with one peptide for a period of 2 days, followed by the application of the other peptide for a subsequent 4-day treatment (Figure 5C). As a result, we observed that leaves treated with SCOOP12 for 2 days prior to the application of SCOOP10 exhibited a senescence phenotype similar to the mock treatment, in contrast to the significant senescence phenotypes displayed in leaves treated with SCOOP10 alone (Figures 5D and 5E). However, when detached leaves were pretreated with SCOOP10 peptides for 2 days before the application of SCOOP12, they exhibited a delayed senescence phenotype similar to the leaves treated with SCOOP12 alone (Figures 5D and 5E). The results suggest a dominant effect of SCOOP12 over SCOOP10 in the regulation of senescence. Because SCOOP12 and SCOOP10 are both recognized by MIK2, the SCOOP peptides might compete for interaction with the MIK2 receptor and SCOOP12 might have the advantage in this competition. To test this hypothesis, an MST assay was set up to analyze the MIK2 binding competition between MIK2-SCOOP10 and MIK2-SCOOP12 in the presence/absence of SCOOP12 or SCOOP10, respectively (Figure 5F). Here, we show that while SCOOP10 has a limited effect on the MIK2-SCOOP12 complex stability maintenance (K_D_ 1.160 μM), SCOOP12 peptide inhibited the interaction between SCOOP10 and MIK2 with a K_D_ of 279 μM (Figure 5F). It is important to mention that even if SCOOP10 had a stronger affinity for MIK2 (K_D_ 0.753 μM) when compared to SCOOP12 (K_D_ 0.836 μM), the application of SCOOP12, but not that of SCOOP10, can affect SCOOP10’s ability to bind MIK2 (Figure 5F). We validated our observation by performing a MIK2-SCOOP interaction pull-down assay in the presence/absence of increasing concentrations of biotinylated SCOOP10 or SCOOP12 (Figure 5G and Supplemental Figures 6A–6C). In fact, while the signal dependent on the interaction between SCOOP10 and MIK2 decreased as more SCOOP12 was applied (Supplemental Figure 6D), the SCOOP12-MIK2 complex resulted in a stable interaction with SCOOP10 application at the concentrations tested (Figure 5G and Supplemental Figure 6E).

**Figure 6:**
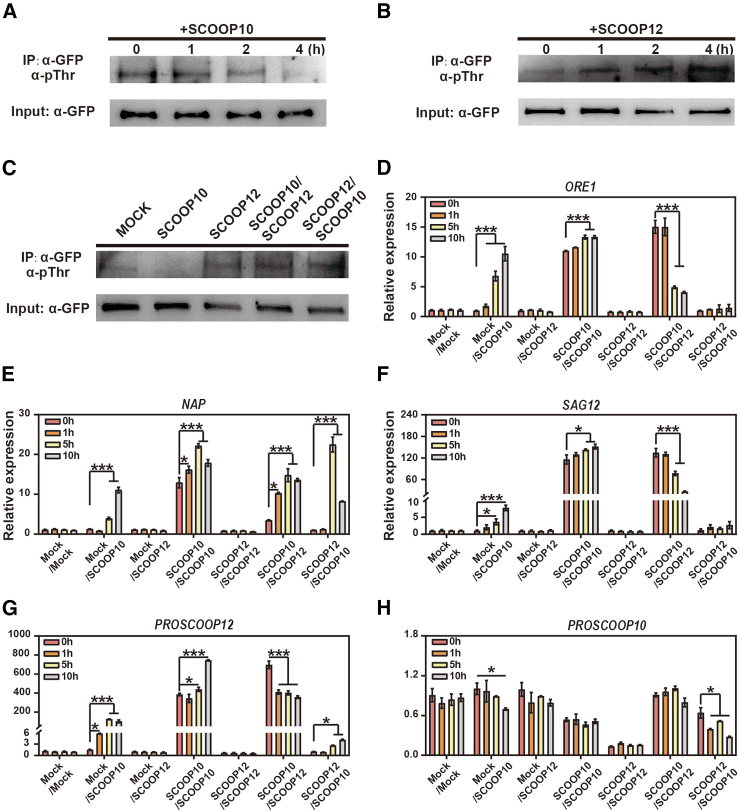
MIK2 phosphorylation is repressed by SCOOP10 application but enhanced by SCOOP12 treatment in *pMIK2:MIK2-GFP* transgenic plants. **(A and B)** The fifth and sixth rosette leaves from 4-week-old *pMIK2::MIK2-GFP/mik2-1* plants were treated with 5 μM SCOOP10 **(A)** or 5 μM SCOOP12 **(B)** for 0, 1, 2, and 4 h. Total proteins were extracted and subjected to immunoprecipitation (IP) using anti-GFP agarose beads. The immunoprecipitated proteins were analyzed with an anti-pThr antibody (top) and an anti-GFP antibody for loading control (bottom). **(C)** MIK2 phosphorylation in detached leaves pretreated with either SCOOP10 or SCOOP12 peptides for 4 h, followed by treatment with the opposite peptide (SCOOP12 or SCOOP10) for an additional 4 h. The immunoprecipitated proteins were analyzed with an anti-pThr antibody (top) and an anti-GFP antibody for loading control. **(D–H)** The expression levels of senescence-associated genes *ORE1***(D)**, *SAG12***(E)**, *NAP***(F)**, *PROSCOOP10***(G)**, and *PROSCOOP12***(H)** were measured under different sequential treatments. The sixth rosette leaves from 1-month-old Arabidopsis plants were pretreated with or without 1 μM SCOOP10 or SCOOP12 peptides, followed by short-term treatments for 0, 1, 5, and 10 h, as indicated in the figures. Bars represent means ± SDs from three independent experiments (*n* = 3 leaves per replicate). Asterisks indicate statistically significant differences compared to the control (0 h), as determined by one-way ANOVA (α = 0.05), followed by Bonferroni correction for multiple comparisons (∗*p* ≤ 0.05; ∗∗∗*p* ≤ 0.001).

Next, we used 4-week-old adult homozygous transgenic lines expressing *pMIK2:MIK2* fused with GFP, in the *mik2-1* background to detect MIK2 kinase phosphorylation *in vivo* in response to peptide treatments. Plants were grown for 4 weeks, and the fifth and sixth leaves were collected and incubated for 0, 1, 2, or 4 h with either SCOOP10 (Figure 6A) or SCOOP12 (Figure 6B) peptides. Following total protein extraction and GFP pull-down, we observed that MIK2 phosphorylation decreased drastically over time compared to the mock controls upon SCOOP10 application (Figure 6A). In contrast, MIK2 showed increased phosphorylation patterns in response to SCOOP12 application (Figure 6B). To better understand the combinatorial effects of both peptides on the regulation of MIK2 kinase phosphorylation, we incubated *pMIK2:MIK2-*GFP leaves for 4 h with either SCOOP10 or SCOOP12. After this initial treatment, the opposite peptides were applied (SCOOP10+SCOOP12 or SCOOP12+SCOOP10), and leaves were sampled after another 4 h. The results shows that while 4 h of SCOOP10 treatment inhibited MIK2 kinase phosphorylation, SCOOP12 induced it as presented in Figures 6A and 6B, in the combination treatment, SCOOP12 was able to outcompete SCOOP10 inhibition effect, triggering MIK2 phosphorylation independently of SCOOP10 presence (Figure 6C). We therefore investigated whether the exogenous application of SCOOP12 and SCOOP10 alone or pretreatments with SCOOP10 or SCOOP12 followed by SCOOP12 or SCOOP10 treatments (SCOOP10/SCOOP12, SCOOP12/SCOOP10), would alter the expression profiling of senescence regulators *ORE1* and *NAP* and senescence marker gene *SAG12* over time. Detached leaves were pretreated with or without SCOOP10 or SCOOP12 for 24 h followed by incubation with mock (droplet digital H_2O [ddH_2O]__), SCOOP10, or SCOOP12 for 0, 1, 5, and 10 h. We observed that while SCOOP10 strongly and relatively quickly induced the expression of the tested transcripts, SCOOP12 had a limited effect compared to the mock controls. When SCOOP12 was applied to SCOOP10-pretreated leaves, a strong inhibition of the SCOOP10-dependent induction of senescence genes was observed, likely supporting the observation that SCOOP12 could outcompete SCOOP10 (1) in its binding ability to MIK2 and (2) in the regulation of the senescence signaling, likely through MIK2 phosphorylation. Conversely, pretreatment with SCOOP12 followed by SCOOP10 treatment inhibited the SCOOP10-dependent induction of both *ORE1* and *SAG12* but did not block the increased expression of *NAP*, suggesting that SCOOP12 might selectively impair only part of the SCOOP10-mediated senescence signaling (Figures 6D–6F). This may indicate that the inhibition of *NAP* is not solely dependent on MIK2 deactivation. In addition, the application of exogenous SCOOP10 peptides was found to induce the expression of *PROSCOOP12* within 1 h from the application of SCOOP10 peptide (Figure 6G). This induction was even stronger when leaves were pretreated with SCOOP10 and treated with SCOOP10 for 1, 5, and 10 h. Interestingly while SCOOP12 treatment alone did not have a significant effect on the regulation of the *PROSCOOP12* levels compared to the mock controls, the effect of SCOOP10 on triggering the induction of *PROSCOOP12* was alleviated by treatment with SCOOP12, likely suggesting that SCOOP12 presence not only can affect the SCOOOP10-MIK2 complex stability/formation but also might compete with the SCOOP10-dependent transcript regulations (Figure 6G). However, the expression of *PROSCOOP10* was found to be sensitive to both SCOOP12 and SCOOP10 application, resulting in reduced expression, which was more pronounced in SCOOP10/SCOOP10-pretreated leaves when compared to the mock controls (Figure 6H). However, *PROSCOOP10* appeared to be strongly regulated by SCOOP12 pretreatment, suggesting that altering the expression of *PROSCOOP10* through the signaling mediated by SCOOP12 would require more time than the effect of SCOOP10 on *PROSCOOP12* regulation. Taken together, our results suggest the presence of a negative feedback mechanism, where SCOOP10 and SCOOP12 regulate each other as potential modulators of the signaling pathway, fine-tuning senescence progression.

## Discussion

As a genetically regulated age-dependent process, leaf senescence involves signaling events that are triggered by various endogenous and environmental factors. The integration of these factors determines the progression of the “senescence syndrome” (Guo, 2013; Ahmad and Guo, 2019). In this study, we discovered a novel senescence-regulating signaling module involving the receptor-like kinase MIK2 and two of the SCOOP peptides (SCOOP10 and SCOOP12). MIK2 is a member of the subfamily XI of LRR RLKs and was first identified to control pollen tube guidance and plant fertilization by cooperating with MIK1 and MALE DISCOVERER1 for recognition of the peptide LURE1 (Li and Yang, 2016; Higashiyama and Yang, 2017). Later, through a genome-wide association study screening, MIK2 was associated with salt (NaCl) stress responses. *Arabidopsis* ecotypes displaying an increased expression of *MIK2* (or *LRR-KISS*) were characterized by enhanced tolerance to NaCl stress, while knockdown line HR-5 with a reduced expression of *MIK2* displayed increased NaCl susceptibility (Julkowska et al., 2016; Yang et al., 2023). Here, we show that *PROSCOOP10* and *PROSCOOP12* transcripts were found to be differently regulated by abiotic stress. *PROSCOOP10* was found to be significantly up-regulated in response to NaCl, drought, and dark treatments, while *PROSCOOP12* seemed to be less responsive to both drought and darkness and strongly responsive to NaCl application (Supplemental Figure 7).

MIK2 was also linked to cell wall integrity (Van der Does et al., 2017; Coleman et al., 2020) and disease resistance (Van der Does et al., 2017). MIK2 was reported to function in elicitor perception in response to infection by *Fusarium* spp. fungi (Van der Does et al., 2017; Coleman et al., 2020). It was found that exogenous peptides corresponding to *Fusarium*-derived SCOOP-like motifs could trigger MIK2-dependent immunity against *Fusarium*. MIK2 was thus suggested as a receptor of SCOOP family members in *Arabidopsis* and was shown to interact directly with SCOOP12. Loss-of-function in *proscoop12* or *mik2* impaired plant resistance against pathogens and *mik2* was insensitive to the SCOOP12-dependent signaling activation (Hou et al., 2021b). In this study, we uncovered the regulatory role of MIK2 during leaf senescence. Similar to its involvement in stress responses, increased expression of *MIK2* leads to a delay in senescence, while *mik2* mutant plants exhibit premature leaf senescence (Figure 1). Because MIK2 has been previously implicated in responses to abiotic stresses (Julkowska et al., 2016) and pathogen infection (Van der Does et al., 2017), it is plausible that MIK2 serves as a critical hub in mediating signaling pathways triggered by diverse physiological factors, likely affecting plant senescence. Similarly small peptides have been characterized to function in various developmental and stress response processes, usually through receptor-like kinases (Engelsdorf et al., 2018). In most cases binding of the peptide ligands affects the downstream signaling in a positive or negative manner (Okuda, 2021). Here, we report that SCOOP10 and SCOOP12 peptide treatments caused the opposite phenotype of leaf senescence in a MIK2-dependent manner (Figures 3 and 4). It is possible that in the SCOOP10/SCOOP12-MIK2 regulatory pathway described in this study, *PROSCOOPs* are expressed during different stages of senescence, likely controlling senescence in a temporal manner. In fact, we found that *PROSCOOP10* is highly expressed during the early stages of senescence (Supplemental Figure 2), likely promoting the initiation of senescence; but as senescence progresses, SCOOP10 signaling triggers the expression of *PROSCOOP12*, likely altering its progression (Supplemental Figure 2). This sequential activation of SCOOP12 suggests a dynamic interplay between SCOOP10 and SCOOP12, mediated by MIK2, as an intricate regulatory mechanism during leaf senescence. However, both peptides have been described to positively regulate MIK2 in immunity and growth inhibition (Hou et al., 2021b; Rhodes et al., 2021), complicating the interpretation of our results. Here, we report that in leaves, SCOOP10 and 12 peptides exert opposite effects on MIK2 phosphorylation, potentially leading to divergent downstream signals. This could occur either through direct modulation of the signaling cascade via distinct phosphorylation patterns on MIK2 or indirectly by altering the composition and formation of receptor complexes (between MIK2-SCOOP and other putative [co]-receptors), which in turn may differentially regulate the progression of leaf senescence. It is important to highlight the cellular context and the specific tissue type (leaves) of our study, which may introduce unique factors that influence how SCOOP10 and SCOOP12 interact with MIK2 and subsequently affect cellular processes like senescence. Peptide ligands from the same family have been reported to be able to bind to the same receptor leading to opposite responses (Guo et al., 2010; Rhodes et al., 2021). The identification of the MIK2 complex through pull-down assays in response to different SCOOP treatments and at various stages of senescence in leaves will be the next step in understanding how SCOOP10/12-MIK2 exert opposite effects on senescence. It will be essential to determine whether changes in its phosphorylation directly influence downstream signaling or if the recruitment of different proteins into the complex varies in response to distinct phosphorylation patterns.

In this study, we show that the binding of SCOOP10 peptides to MIK2 can be displaced by SCOOP12 (Figure 5G and Supplemental Figure 6) even if, surprisingly, SCOOP10 was found to have a stronger affinity binding ability (K_D_ 0.753 μM) for MIK2 when compared to that of SCOOP12 (K_D_ 0.836 μM) (Figure 5F). We hypothesized that during early senescence events, SCOOP10 serves as a starting senescence signal. As senescence progresses, SCOOP12 becomes the dominant peptide that binds to MIK2, likely preventing senescence from progressing too quickly. Both SOMATIC EMBRYOGENESIS RECEPTOR KINASE 3 (SERK3) and SERK4 have been suggested to act as MIK2 co-receptors, subsequently requiring cytoplasmic signaling events involving BOTRYTIS-INDUCED KINASE 1 (BIK1) and AVRPPHB SUSCEPTIBLE1-LIKE 1 (PBL1) (Hou et al., 2021b). It has been shown that when SCOOP12 binds to MIK2, it forms a complex with BCL2 ANTAGONIST/KILLER 1 (BAK1) and SERK4 (Hou et al., 2021b). However, while MIK2 loss-of-function mutants are completely insensitive to SCOOP application, *bak1* mutants seem to be required for the SCOOP12-induced root growth inhibition; however, it is sensitive to SCOOP10 application (Hou et al., 2021b), likely suggesting the involvement of other co-receptors for MIK2 in mediating the responses to other SCOOPs. The redundancy between BAK1 and SERK4 was evidenced by the nearly abolished SCOOP12-mediated root growth inhibition in the *bak1-5 serk4* double mutant, although it showed a milder phenotype in response to SCOOP10 treatment (Hou et al., 2021b). These observations led us to hypothesize that even in the absence of the key co-receptors, SCOOP10 responses, which might be dependent on MIK2 inactivation, are not fully abolished, suggesting that MIK2 in combination with several (co-)receptors might execute its function. Previous studies indicate that senescence upregulates *SERK4* expression, and the *serk4-1* knockout mutant exhibits early leaf senescence akin to the *mik2* mutant (Li et al., 2019), implying a role for both MIK2 and SERK4 in senescence regulation in *Arabidopsis*. It was suggested that SCOOP/MIK2 activates BIK1 and PBL1 (Hou et al., 2021b). However, *bik1 pbl1* double mutants show only mild alleviation of the SCOOP10-induced root growth inhibition (Hou et al., 2021b), suggesting that the complex formed in response to MIK2 and SCOOP10 binding might recruit proteins different from BIK1/PBL1 to regulate root growth. It has been reported that the *Catharanthus roseus* RLK FERONIA (FER), similarly to MIK2, negatively controls NaCl stress responses (Feng et al., 2018; Gigli-Bisceglia and Testerink, 2021; Zhao et al., 2020; Gigli-Bisceglia et al., 2022). Moreover FER acts as a receptor for the RAPID ALKALINIZATION FACTOR (RALF) peptides and modulates receptor kinase complex assembly during pathogen-triggered immunity (PTI) (Stegmann et al., 2017). FER promotes the assembly of the receptor complex EF-Tu Receptor (EFR)/Flagellin-Sensing 2 (FLS2)-BAK1 in response to elf18/flg22. However, while RALF23 perception by FER suppresses the formation of the EFR/FLS2-BAK1 complex, potentially dampening PTI signaling, RALF17 enhances the assembly of the FER-mediated EFR-BAK1 complex by competing with RALF23 for binding to FER (Stegmann et al., 2017). A similar scaffolding mechanism may be present in response to MIK2-SCOOP binding. Further investigation will be essential to uncover this hypothesis. Other antagonistic regulations, independent of PTI regulations, have been reported recently for the RALF peptide family, highlighting the opposite role of peptides in binding to the same receptor. For example, during pollination, RALF4/19 and RALF34 antagonistically regulate pollen tip integrity to avoid premature burst (Zhang et al., 2023). RALF23 and RALF33 peptides compete with the POLLEN COAT PROTEIN B-class peptides in binding to the *C. roseus* RLKs ANJEA-FERONIA (ANJ–FER) receptor complex, which functions in regulating reactive oxygen species production during pollen hydration (Liu et al., 2021).

**Figure 7:**
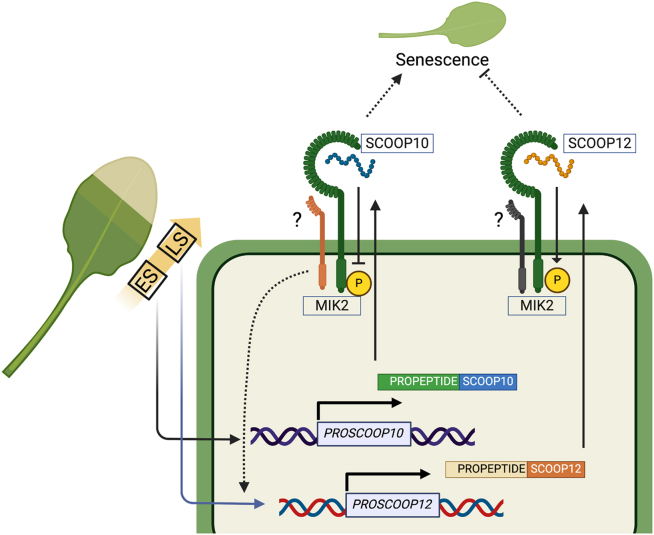
A proposed model illustrates the possible mechanism through which SCOOP10 and SCOOP12 regulate leaf senescence via competitive interaction with MIK2. The transcriptional activation of *PROSCOOP10* and *PROSCOOP12* is regulated by age. During the early stages of leaf senescence, *PROSCOOP10* accumulates, and the binding of SCOOP10 inhibits MIK2 phosphorylation and function, which in turn triggers leaf senescence. SCOOP10 recognition enhances the expression of *PROSCOOP12*, likely resulting in increased SCOOP12 synthesis, increased MIK2 phosphorylation, and consequent inhibition of leaf senescence. ES, early senescent leaf; LS, late senescent leaf.

Here, we hypothesize that the sequential and antagonistic interactions between SCOOP10-MIK2 and SCOOP12-MIK2, which impact MIK2 phosphorylation, alter its functionality. These changes in MIK2 phosphorylation states, triggered by the recognition of different SCOOPs, create a knot-like structure that ensures the timely progression of leaf senescence (schematized in Figure 7). In this sense, the MIK2-mediated senescence pathway triggered by SCOOP12 might act as a safeguard, preventing senescence from progressing too rapidly and ensuring the orderly progression of nutrient recycling. The SCOOP-MIK2 module represents a novel mechanism in plant senescence, where antagonistic peptides compete for the same receptor to fine-tune the control of leaf senescence. In our study we also note that in addition to SCOOP10 and SCOOP12, treatments with several other SCOOP peptides caused altered senescence phenotypes (Supplemental Figure 3). More profound senescence was induced by SCOOP10 treatments in WT than in *mik2* mutant plants (Figures 3A–3C), suggesting that in addition to the SCOOP10/12-MIK2 regulatory module, more complex interactions between SCOOP peptides and membranes receptors might be involved in the regulation of leaf senescence. Our discovery highlights the intricate and dynamic nature of peptide signaling in plant development and provides insights into the regulatory networks controlling leaf senescence.

## Methods

### Plant growth and detached leaf treatments

*A. thaliana* ecotype Columbia-0 and mutants used in this study include *mik2-1* (SALK_06269), *mik2-2* (CS419958), *proscoop10-1* (SALK_080439), and *proscoop10-2* (SALK_027949), which were obtained from ABRC. The *proscoop12* CRISPR-Cas9-generated mutants were kindly provided by Dr. Elia Stahi at the University of Lausanne, Switzerland (Stahl et al., 2022). Imbibed seeds were incubated at 4°C in a refrigerator for 2 days before sowing on the soil mix (3 parts commercial soil: 1 parts vermiculite) and grown under the long-day condition (16 h light/8 h dark) in a growth chamber with 150 μmol m^−2^ s^−1^ light intensity. For the senescence phenotyping of detached leaves, the fifth and sixth rosette leaves of 25-day-old plants were removed and placed in Petri dishes with the adaxial side facing up. The Petri dishes were sealed with parafilm to prevent water loss, and the leaves were incubated with deionized water. The application of exogenous peptides followed the previously established method (Zhang et al., 2022a, 2022b). For dark-induced leaf senescence assays, detached rosette leaves were incubated in deionized water placed in the dark condition for 6 days. At least three biological replicates were included for each assay. For senescence phenotype analysis under normal growth conditions, homozygous plants were grown alongside Col-0. All data including plant phenotypes and gene expression presented in this study were analyzed with GraphPad Prism 9, and at least three biological replicates were included in each experiment. Chl content, photochemical efficiency of PSII (Fv/Fm ratio), and ion leakage were determined as previously described (Zhang et al., 2020, 2022b; Guo et al., 2022).

### Generation of transgenic plants

The 2200-bp *MIK2* promoter (*pMIK2*) and the 2000-bp *SAG12* promoter (*pSAG12*) were amplified from Col-0 genomic DNA. The coding sequences (CDSs) of *MIK2*, *PROSCOOP10*, and *PROSCOOP12* were cloned via reverse-transcription PCR (RT-PCR). The cloned fragments were inserted into the pEASY-Blunt vector (TransGen Biotech) to generate sub-clone vectors *pMIK2*-Blunt, *pSAG2*-Blunt, *gMIK2*-Blunt, *gSCOOP10*-Blunt, and *gSCOOP12*-Blunt, which were confirmed by sequencing. For the complementation test, *pMIK2* and *MIK2* CDSs from *pMIK2* and *gMIK2*-Blunt were inserted into the *pPZP211* vector separately at the *EcoRI* and *SacI* sites to generate the *pMIK2:MIK2* construct by Infusion (Vazyme Biotech). For *SAG12*-induced expression, *pSAG12* and the CDSs of *MIK2*, *PROSCOOP10*, and *PROSCOOP12* were cloned into *pPZP211* to obtain *pSAG12:MIK2*, *pSAG12:PROSCOOP10*, and *pSAG12:PROSCOOP12*. For subcellular localization assays, the *MIK2* CDSs without stop codon fused with GFP was cloned into pCHF_3_ to generate *pCHF3::MIK2-GFP* vector by Infusion upon digestion at the *Sac I* site. For the generation of the *pMIK2::MIK2-GFP* vector, the sequence of *pMIK2* and *MIK2-GFP* were amplified using *pMIK2*-Blunt and pCHF3MIK2-GFP vectors as the template and then were inserted into the pZP211 backbone. All primers used in this study were synthesized by Personal Biotechnology Company, and the sequences are listed in Supplemental Table 2. The recombinant vectors described above were transformed into *Agrobacterium tumefaciens* strain GV3101 for subsequent transformation of *Arabidopsis* plants via the floral dipping method. For the screening for transgenic plants, seeds were surface sterilized with 75% ethanol for 5 min, washed with 100% ethanol for 1 min, and sown on half-strength Murashige and Skoog medium containing appropriate antibiotics, pH 5.8, followed by 2 days’ incubation in the dark at 4°C. Ten days after germination, plants were transplanted to the soil mix in the growth chamber and grown under the conditions described above. Genotyping analyses were performed with leaf tissues from plants grown in soil using gene-specific primers (Supplemental Table 2).

### Gene expression analysis

Total RNA was isolated from *Arabidopsis* leaves with the RNAiso Plus (TAKARA) and reverse transcribed using the PrimeScript RT reagent Kit (TAKARA). qPCR reactions were implemented in an Applied Biosystems 7500 Real-Time PCR system with the SYBR Premix Ex Taq II (TAKARA). *ACTIN* was used as the reference gene for qPCR analyses. The primers used for qPCR are listed in Supplemental Table 2.

### Protein expression and purification

To express MIK2 protein in the soluble form, the truncated sequence of MIK2 (MIK2 ^LRR^) expressing only the ectodomain (residues 45–707) was amplified from the *gMIK2*-Blunt subclone vector and inserted into the *pET41* vector, fused with a 6× His-MBP tag. Then, the ectodomain of MIK2 (MIK2^LRR^) in the absence of the putative N-terminal signal peptide was expressed in *E. coli* strain BL21 (TransGen). Cell cultures were grown to 0.6 optical density 600 and then induced with 0.3 mM isopropyl-β-d-thiogalactopyranoside for 30 h at 13°C. The mixture was then centrifuged at 12 000 rpm to separate the cells from the medium. The pellets were resuspended with the binding buffer (20 mM phosphate buffer, 500 mM NaCl, 50 mM imidazole, pH 7.4). The cells were sonicated on ice for 30 min in the lysis buffer (0.2 mg/mL lysozyme, 20 μg/mL DNAse, 1 mM MgCl_2_, 1 mM PMSF). After centrifugation (12 000 rpm for 30 min at 4°C), the supernatant was collected for purification. His-MBP-tagged MIK2 ^LRR^ isolation was performed using the Mag-Beads His-Tag Protein Purification Kit (Sangon Biotech) following the instructions of the manufacturer.

### MST analysis

A Monolith NT.115 system (Nanotemper Technologies) was used to perform MST assays. To measure the binding affinity with MIK2^LRR^, SCOOP10 and SCOOP12 peptides were labeled with fluorescent 5-FAM at the N termini (GenScript), and the final working concentration of labeled peptides was adjusted to 0.05 μM with ddH_2_O. The concentration of purified MIK2^LRR^ was measured with the BCA Protein Quantification Kit (Vazyme) and then diluted with ddH_2_O to 20 μM. To analyze the binding affinity between 5-FAM-SCOOP10/12 and MIK2^LRR^, the purified MIK2^LRR^ was incubated with FAM-SCOOP10 or FAM-SCOOP12 for 10 min at room temperature and then loaded into silica capillaries (Monolith NT.115 Standard Treated Capillaries, MO-K002). MST assays were performed according to protocols provided by NanoTemper Technologies (20% light-emitting diode [LED] power and 40% MST power). MST results were analyzed using the Nanotemper analysis software (MO. Affinity) (Jerabek-Willemsen et al., 2011). The competitive binding analysis were performed as described previously (Blech et al., 2013; Liu et al., 2021). To analyze the competitive binding between SCOOP12 and SCOOP10 to MIK2, 0.5 μM FAM-SCOOP10 peptide and 15 μM purified MIK2^LRR^ were mixed and incubated for 10 min at room temperature in the dark. A range of concentrations of SCOOP12 peptides were introduced to the FAM-SCOOP10/MIK2 LRR mixture, and the mixture was co-incubated for 10 min. The mixtures were subsequently loaded into silica capillaries to conduct MST assays using 20% LED power and 20% MST power. The dissociation constant K_i_ was determined using the Ki Finder Tool available on the NanoTemper Technologies website (http://www.nanotempertechnologies.com/get-it-all/tools/ki-finder/).

### Pull-down assay

To investigate the interaction between SCOOP10 or SCOOP12 and MIK2, the purified MIK2^ECD^ was combined with biotinylated SCOOP10 or SCOOP12 peptides in 500 μL of interactive buffer consisting of 20 mM Tris-HCl, pH 7.3, 150 mM NaCl, 0.1% NP-40) and incubated at 4°C for 1 h. Subsequently, 30 μL streptavidin magnetic beads (New England Biolabs) were added to each sample and incubated for 1 h at 4°C, followed by washing 5–6 times with 1.0 mL of interactive buffer. The proteins bound to the beads were then eluted using interactive buffer containing 5× SDS loading buffer, and western blot analysis was conducted as previously described (Lan et al., 2023). In the competitive binding assay, 50 nM purified MIK2 protein featuring a His-tag at its C-terminal was initially mixed with 250 nM of biotinylated SCOOP10. Following this, a range of concentrations of SCOOP12 were introduced in the specified pull-down buffer, as outlined earlier. After thorough mixing, the beads underwent 5–6 washes to meticulously remove any residual proteins, and then the preparations were subjected to detailed analysis via western blotting to probe the interactions.

### MIK2-phosphorylation assay

Transgenic *A. thaliana* T3 homozygous plants expressing MIK2-GFP under the control of its native promoter were grown under long-day conditions (16 h light/8 h dark) for 4 weeks before sampling. The fifth and sixth rosette leaves from *pMIK2::MIK2-GFP/mik2-1* plants were treated with either 5 μM SCOOP10 or 5 μM SCOOP12 for 0, 1, 2, and 4 h. To evaluate the competition between SCOOP peptides, detached leaves were pretreated with either 5 μM SCOOP10 or 5 μM SCOOP12 for 4 h, followed by treatment with the alternate peptide (SCOOP12 or SCOOP10) for an additional 4 h. Leaves were subjected to vacuum infiltration at room temperature for 30 min. Following treatment, the leaves were rapidly frozen in liquid nitrogen and ground into a fine powder. For total protein extraction, ground tissue was suspended in extraction buffer (150 mM Tris-HCl, pH 7.5, 150 mM NaCl, 10% glycerol, 10 mM EDTA, 1 mM Na₂MoO₄, 1 mM NaF, 10 mM dithiothreitol, 1% IGEPAL, 1% protease inhibitor cocktail [Roche, 04693132001], 5% phosphatase inhibitor cocktail [Roche, 04906845001]) at a ratio of 1:2 (w/v) tissue to buffer. The samples were incubated for 30 min at 4°C with gentle agitation, followed by centrifugation at 14 000 rpm for 20 min at 4°C to remove debris. The supernatant was then incubated with anti-GFP agarose beads (Abcam, AE079) overnight at 4°C for immunoprecipitation (IP). The beads were washed five times with IP buffer (150 mM Tris-HCl, pH 7.5, 150 mM NaCl, 10% glycerol, 10 mM EDTA, 1 mM Na₂MoO₄, 1 mM NaF), and bound proteins were eluted by adding 5× SDS loading buffer and heating at 95°C for 15 min. The 30-μL eluted proteins were resolved on 7.5% SDS-PAGE gels and transferred onto polyvinylidene fluoride membranes. Immunoblotting was performed to detect MIK2 using anti-GFP (1:5000; Abcam, AB6556), goat anti-rabbit immunoglobulin G (IgG) (H + L)-horseradish peroxidase (HRP) antibody (1:4000; Abcam, AB6721) and its phosphorylation status using anti-pThr antibodies (1:750, Cell Signaling, 9386), goat anti-mouse IgG (H + L)-HRP antibodies (1:2000; Abcam, AB6789), respectively.

### Peptides synthesis

All peptides used in this study were synthesized by GenScript with >98% high-performance liquid chromatography purity. The peptide sequences are listed in Supplemental Table 3 referring to previously published papers (Hou et al., 2021b; Rhodes et al., 2021).

### Confocal microscopic analysis

To assess protein expression in leaves, MIK2 fused to GFP under the control of the 35S promoter was transiently expressed in *Nicotiana benthamiana* leaves. Imaging was performed using a confocal microscope (TCS-SP8, Leica) with a 488-nm laser for excitation. To examine the subcellular localization of MIK2 in roots, 5-day-old seedlings expressing *MIK2-GFP* were stained with 2 μM FM4-64 for 5 min. Epidermal cells of these seedlings were then imaged using the same SP8 confocal microscope. The GFP and FM4-64 fluorophores were excited with lasers at 488 and 556 nm, respectively. Emissions were simultaneously collected in the ranges of 500–530 nm for GFP and 580–650 nm for FM4-64. All images were processed using the Leica Application Suite X (LAS X) software to enhance visualization of the data.

### Statistical analysis

Statistical significance was assessed using either Student’s t test or one-way ANOVA, followed by Bonferroni correction. Statistical details of the experiments are specified in the figure legends. All statistical analyses were performed in GraphPad Prism 10.

## Funding

We thank Dr. Elia Stahi at the University of Lausanne and ABRC for kindly providing *Arabidopsis* mutants. This work was supported by the Agricultural Science and Technology Innovation Program, Chinese Academy of Agricultural Sciences (ASTIP-TRI02 to Y.G.), the 10.13039/501100001809National Natural Science Foundation of China (32270332 to Y.G., 32370337 to J.W. and 31970204 to W.L.), and European Research Council (ERC) under the EU Horizon 2020 Research and Innovation Programme (grant agreement 724321 to C.T.). Z.Z. was supported by the Graduate School of Chinese Academy of Agricultural Sciences and the Wageningen University Joint PhD Programme.

## Acknowledgments

No conflict of interest declared.

## Author contributions

Y.G. and N.G.-B. conceptualized and designed the study. Y.G., C.T., N.G.-B., and W.L. supervised Z.Z., who performed and collected all the data presented in this manuscript. Z.Z. and N.G.-B. drafted the manuscript, and Y.G., C.T., and N.G.-B. edited the manuscript. S.L. and J.L. contributed to the protein expression and interaction assays. J.W. performed the data analysis and figure preparation. All authors critically reviewed the manuscript and approved it for publication.

## Supplemental information

Document S1. Supplemental Figures 1–7 and Supplemental Tables 1–3

Document S2. Article plus supplemental information
