# Supplementary material for: SCOOP10 and SCOOP12 peptides act through MIK2 receptor-like kinase to antagonistically regulate Arabidopsis leaf senescence
Source: Mol Plant. 2024 Dec 2;17(12):1805–19. doi: 10.1016/j.molp.2024.10.010 (PMC11630628; doi:10.1016/j.molp.2024.10.010)
Supplement: Document S1. Supplemental Figures 1–7 and Supplemental Tables 1–3 [file mmc1.pdf]

Molecular Plant, Volume *17*

Supplemental information

SCOOP10 and SCOOP12 peptides act through MIK2 receptor-like kinase to antagonistically regulate *Arabidopsis* leaf senescence

Zhenbiao Zhang, Nora Gigli-Bisceglia, Wei Li, Saijie Li, Jie Wang, Junfeng Liu, Christa Testerink, and Yongfeng Guo

Supplemental Information

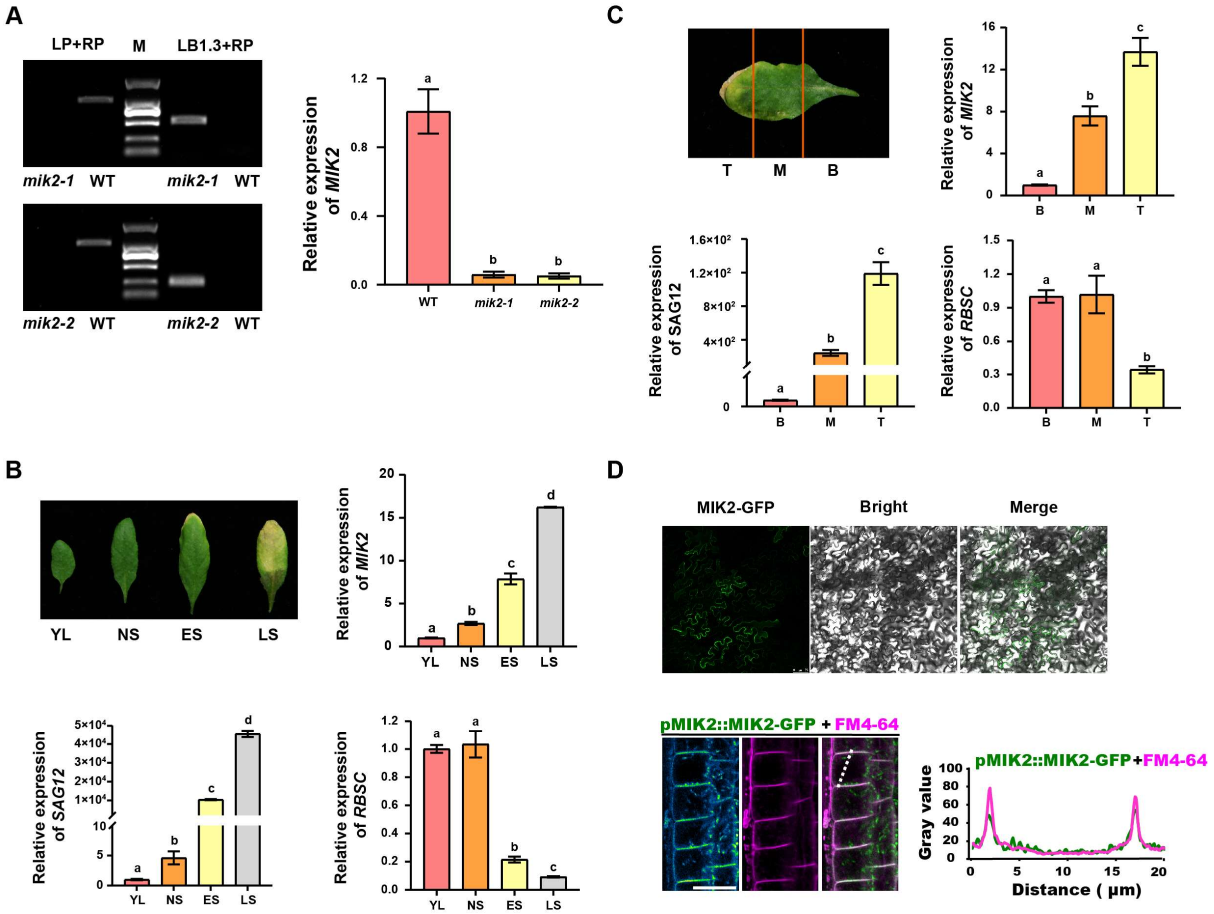

Figure S1. Characterization of the MIK2 loss of function lines and the tagged MIK2-GFP lines. (A) Genotyping of T-DNA insertion mutants (*mik2-1* and *mik2-2*) and analysis of transcript levels using qRT-PCR. (B) Schematic representation of different senescence stages, along with the relative expression levels of *MIK2*, *SAG12*, and *RBCS* determined by qRT-PCR in the indicated leaves. The leaves are categorized as young leaf (YL), fully extended non-senescent leaf (NS), early senescent leaf (ES), and late senescent leaf (LS). The analysis was performed in three independent experiments. (C) Schematic representation of Arabidopsis 6th rosette leaves from 5-week-old plants is presented, along with the expression analysis of *MIK2*, *SAG12*, and *RBCS* in different parts of a leaf, including the base (B), middle (M), and tip (T). The experiment was replicated three times. (D) Subcellular localization of the GFP-MIK2 fusion protein was detected in tobacco leaves (*35S:MIK2-GFP,* upper panel) and in Arabidopsis roots (*pMIK2:MIK2-GFP mik2-1*, lower panel) by using confocal microscopy. GFP-MIK2 co-localizes with the PM dye FM4-64 in *pMIK2:MIK2-GFP mik2-1* roots. The scale bar represents 75 µm. Bars represent means ± SD of three biological replicates. Letters represent statistically significant difference between genotypes (A), age (B) or leaf part (C) as determined by one-way ANOVA (α = 0.05) followed by Bonferroni post-hoc test.

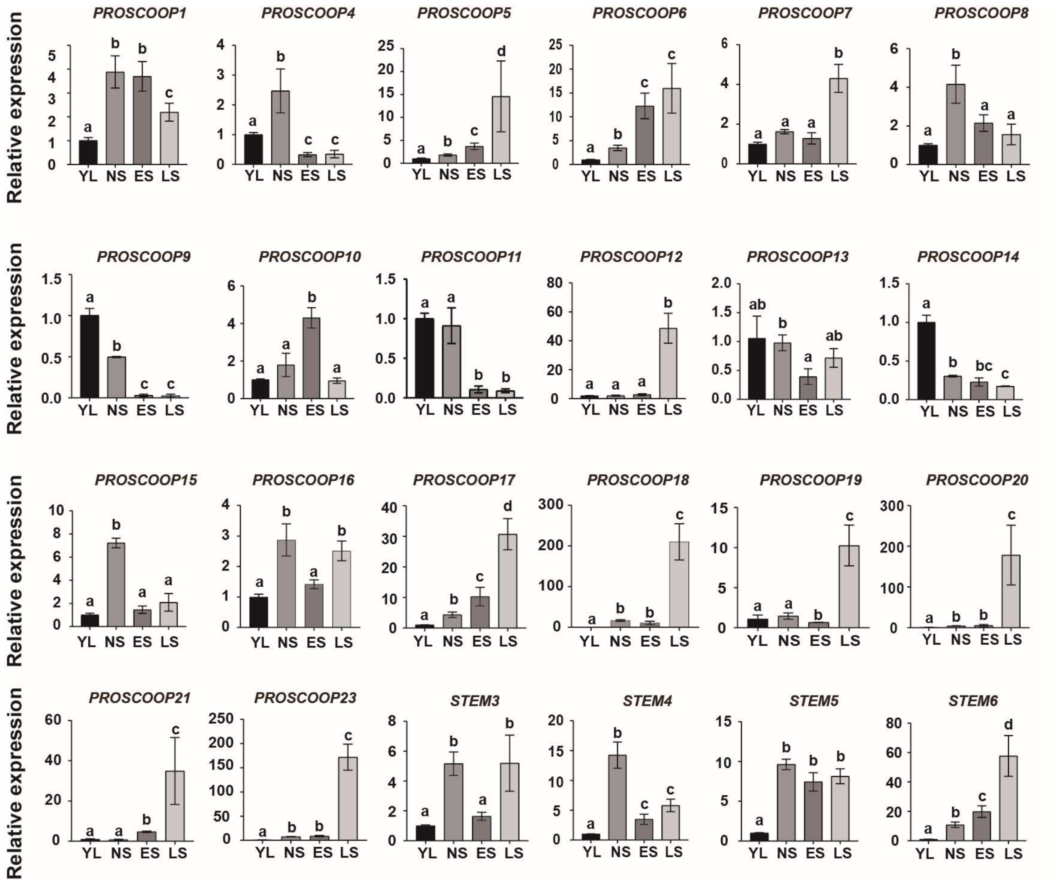

Figure S2. Relative expression of *PROSCOOP*s at different leaf senescence stages. The leaves are categorized as young leaf (YL), fully extended non-senescent leaf (NS), early senescent leaf (ES), and late senescent leaf (LS). Bars represent the means ± SD of three biological replicates. Different letters represent statistically significant difference analyzed by one-way ANOVA analysis (α = 0.05) followed by Bonferroni post-hoc test.

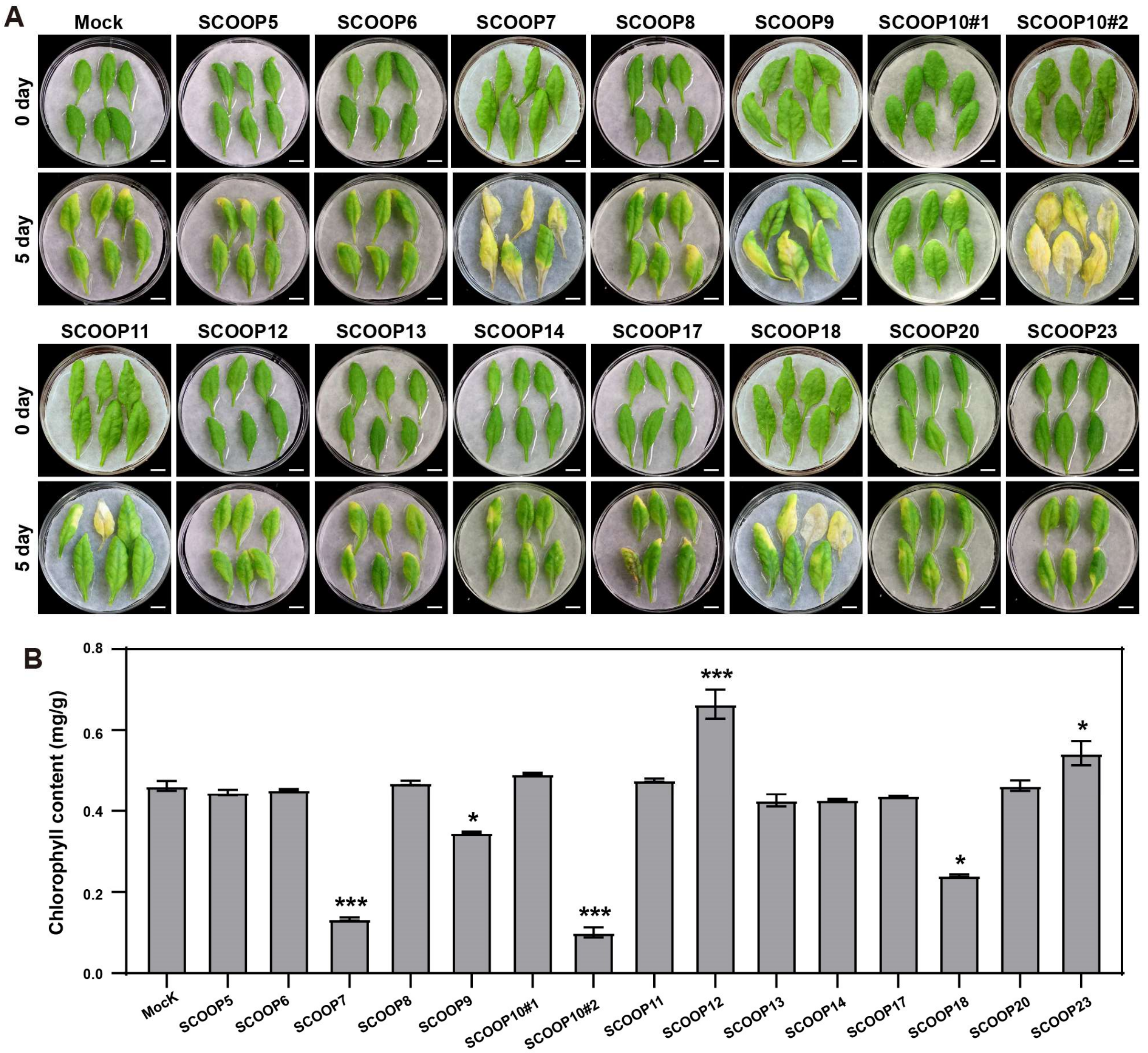

Figure S3. Effect of SCOOP application on senescence progression. (A) Senescence phenotype analyzed in the 5th and 6th rosette leaves from 4-week-old WT plants upon exogenous application of synthetic SCOOP peptides (1 μM). (B) Chl content was measured in detached leaves as shown in (A). Bars represent the means ± SD each containing 6 leaves. Asterisks indicate statistically significant differences compared to mock, as determined by one-way ANOVA (α = 0.05) analysis followed by Bonferroni post-hoc test for multiple comparisons (*P ≤ 0.05, **P ≤ 0.01, ***P ≤ 0.001).

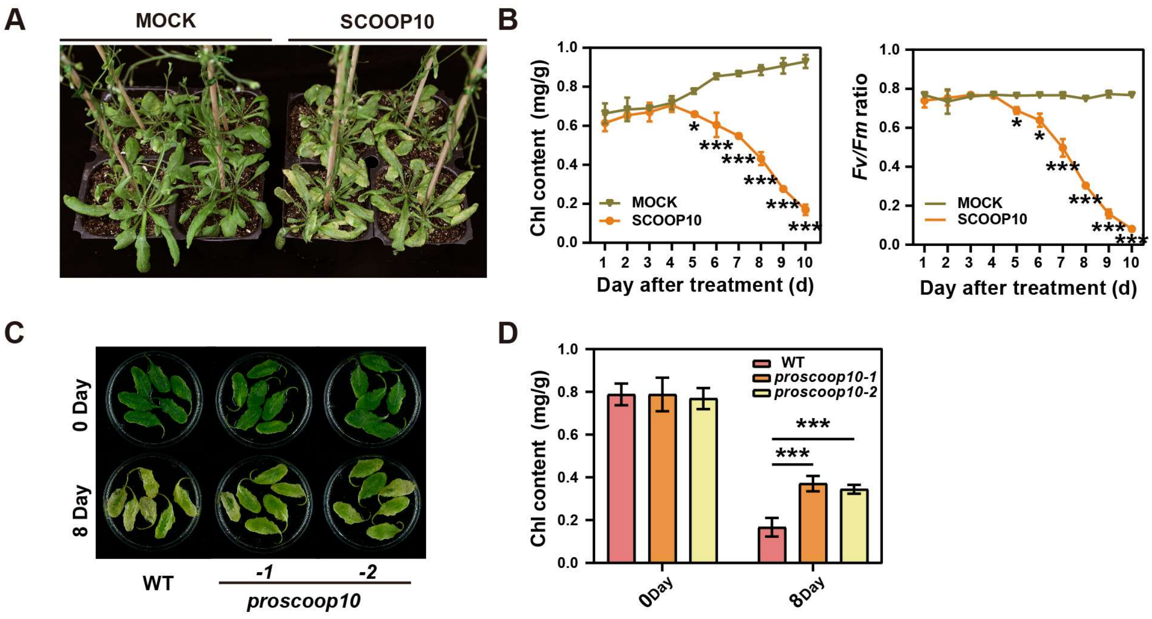

Figure S4. (A) Senescence phenotype of 4-week-old WT plants sprayed with synthetic SCOOP10 peptides (1 μM) or ddH_2O (MOCK) for 10 days. (B) Measurement of Chl content and ion leakage of the 6th rosette leaves from plants shown in (A) after 1 to 10 days of treatments. Three independent experiments were performed with 8 leaves measured for each replicate. Bars represent the means ± SD. Asterisks indicate statistically significant differences compared to MOCK, as determined by one-way ANOVA (α = 0.05) followed by Bonferroni post-hoc test for multiple comparisons (*P ≤ 0.05, ***P ≤ 0.001). (C) Senescence phenotype of the 6th rosette leaves from 4-week-old WT plants and *proscoop10-1*, *proscoop10-2* incubated in ddH_2O upon darkness treatments for 8 days. (D) Chl content measurement of detached leaves present in (C) at indicated time points. Bars represent the mean ± SD of 3 biological replicates each containing 6 leaves. Asterisks indicate statistically significant differences compared to WT, as determined by one-way ANOVA analysis (α = 0.05) followed by Bonferroni post-hoc test for multiple comparisons (***P ≤ 0.001).

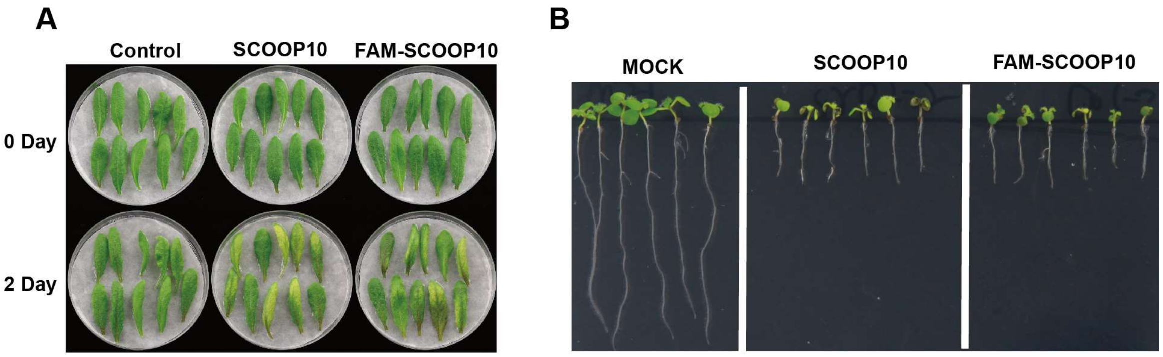

Figure S5. Identification of the bioactivity of fluorescently labeled FAM-SCOOP10 peptides. (A) Leaf senescence was analyzed in rosette leaves from 4-week-old plants treated with FAM- SCOOP10 and SCOOP10 peptides. (B) FAM-SCOOP10 induced root growth inhibition similarly to SCOOP10. 4-day old seedlings were transferred to 1/2 MS plates containing MOCK (ddH_2O), 0.5 µM SCOOP10 or FAM- SCOOP10 peptides and grown for 7 additional days under long-day conditions.

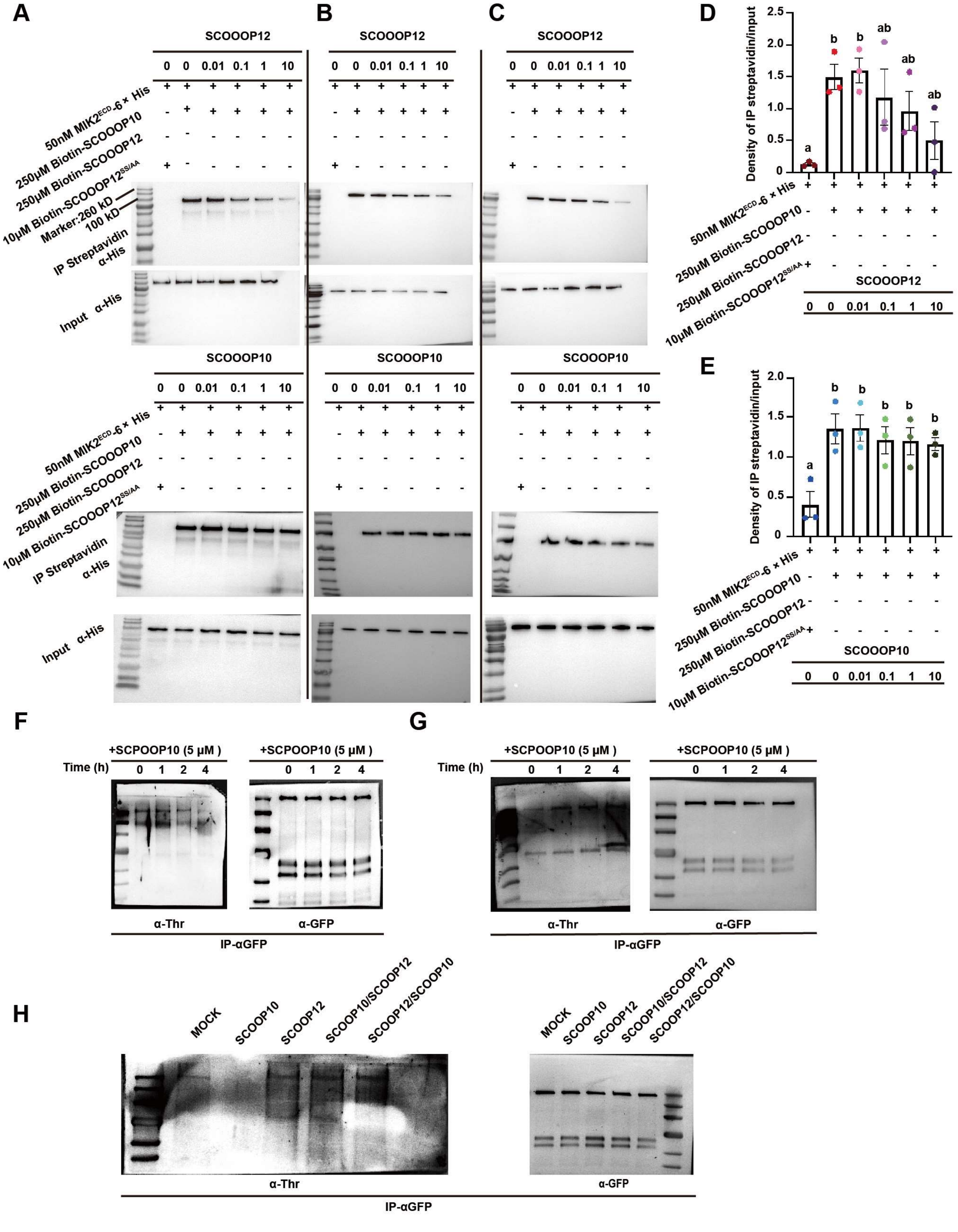

Figure S6. (A) Gels of cutouts presented in Figure 5G together with 2 Independent (B, C) biological replicates of the experiment shown in figure 5G. (D/E) Quantification of Western blot bands by densitometry through FIJI. The band intensity of each lane was plotted after normalization to the input MIK2-His signal. The experiments shown in A, B, and C were pooled, analyzed, and are presented in the same graph as average signal intensity ± SEM. Letters represent statistically significant difference to control (0 μM) based on one-way ANOVA (α = 0.05) and Tukey's HSD. (F/G/H) Gels of cutouts presented in Figure 6 A, B and C.

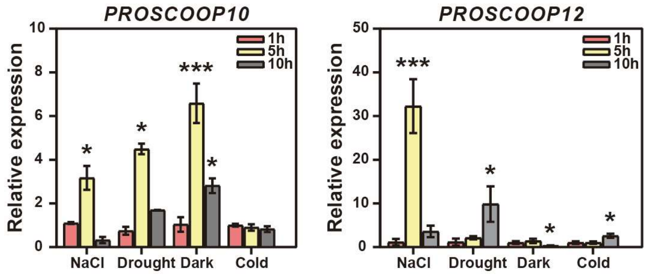

Figure S7. Relative expression of *PROSCOOP10* and *PROSCOOP12* was analyzed in the 6th rosette leaves detached from 4-week-old WT plants after treatments with diverse abiotic stresses (150 mM NaCl, 300 mM mannitol, darkness and 4°C) for 1, 5 and 10 hours. Three biological replicates were included for each experiment. Bars indicate the mean ± SD. Asterisks indicate statistically significant differences compared to control (1 h), as determined by one-way ANOVA (α = 0.05) analysis followed by Bonferroni post-hoc test for multiple comparisons (*P ≤ 0.05, ***P ≤ 0.001).

Supplemental Tables

Gene number	NASC stock number	Gene number	NASC stock number	Gene number	NASC stock number	Gene number	NASC stock number	Gene number	NASC stock number
AT1G72300	N572802	AT2G37050	N571422	AT3G23750	N615534	AT4G30520	N611290	AT5G16000	N560808
	N590983		N643700		N576864	AT4G23740	N561788		N517538
AT5G48380	N508775	AT2G19190	N331648	AT3G25560	N544363		N505132	AT5G45840	N554077
	N528071		N519981		N564245	AT4G28490	N515075	AT5G49760	N502203
AT1G51860	N535831	AT2G45340	N507062	AT3G14840	N540387	AT4G37250	N590416		N618908
	N531400		N611582		N594512		N512265	AT5G46330	N562054
AT1G72300	N572802	AT2G13800	N647275	AT3G13065	N505553				N599605
AT1G51790	N525281		N571635	AT3G28450	N611476	At4g18640	N504879	AT5G58140	N551210
	N525291	AT2G07040	N610661		N611477	At4g20140	N564029		N647745
AT1G27190	N632066		N610653	AT3G02880	N519840		N559157	AT5G58150	N621857
	N610440	AT2G45830	N635799		N540345	At4g20270	N800012		N647745
AT1G53730	N639842		N608106	At3g13380	N538909		N504121	AT5G01890	N555351
	N577702	AT2G01820	N872720	At3g14350	N539120	At4g20940	N472895	AT5G10020	N579994
AT1G31420	N691006	AT2G26730	N540481	At3g17840	N410869	At4g22130	N571743		N625544
	N689965		N646640	At3g19700	N573260	At4g22730	N513143	AT5G10290	N522249
AT1G28440	N604365	AT2G23950	N565680	At3g20190	N406238	At4g28650	N678357	AT5G48655	N331698
	N608127		N571166	At3g24660	N465527		N595005	AT5G01940	N511358
AT1G66150	N506428	AT2G25790	N523303	At3g46330	N426569	At4g29180	N838386		N509439
	N506435		N587435	AT3G28040	N593475	At4g33430	N534523	At5g01950	N421183
AT5G65710	N650495	AT2G35620	N800011		N593189		N662069	At5g05160	N623818
	N579528	AT2G20850	N871886	AT3G08680	N389505	At4g34220	N2103441	At5g06820	N693199
AT1G51890	N687128	AT4G31250	N870012	AT3G02130	N734370	At4g36180	N456867	At5g07180	N326261
AT2G13790	N557955	AT2G31880	N320040	AT3G03770	N309716	AT4G03390	N501389	At5g07280	N843279
	N605409	AT2G19190	N331660	AT3G47090	N730890		N501048	At5g20690	N472094
AT1G79680	N640705	AT2G02220	N800002	AT3G62190	N721570	AT4G08850	N561769	AT5G67280	N544853
	N632887	AT2G33170	N733635	AT3G47570	N734350		N720704		N511465
AT1G17230	N597514	AT2G26330	N26676			AT4G03390	N719224	AT5G01550	N608096
	N601617	At2g01950	N516024			AT4G39400	N312870		N608000
AT1G09970	N583112	At2g04300	N503328			AT4G38130	N370961	AT5G62230	N581669
	N594492	At2g14440	N406451			AT4G03390	N719223		N664725
AT1G67510	N560478	At2g14510	N842997			AT4G39270	N734931	AT5G45800	N307350
	N554094	At2g15300	N518028					AT5G51350	N811971
AT1G72460	N502870	At2g16250	N458408					AT5G20480	N872588
	N504993	At2g19210	N541066					AT5G55590	N812482
AT1G34110	N514736	At2g19230	N571724					AT5G55590	N870218
	N539687	At2g23300	N407247					AT5G65700	N163040
AT1G66830	N623540	At2g24230	N407944					AT5G56040	N800047
	N502631	At2g27060	N26801					AT5G59670	N26554
AT1G52857	N504907	At2g28960	N505444					AT5G63400	N827410

	N504995	At2g28990	N825357					AT5G63410	N827410
AT1G67510	N640207	At2g36570	N877772					AT5G59680	N830868
	N554094	At2g41820	N638018					AT5G25930	N346871
AT1G06840	N634409	At2g42290	N693675						
AT1G51800	N637388	At2g45340	N611584						
AT1G53440	N320961								
AT1G72180	N720568								
AT1G53190	N333113								
AT1G29720	N858177								
AT1G56140	N730971								
AT1G35710	N334877								
AT1G56130	N753123								
AT1G53430	N734378								
AT1G63430	N731642								
AT1G01720	N720586								
AT1G01720	N337650								
AT1G78980	N731551								
AT1G05700	N875161								
AT1G07550	N598074								
At1g07560	N410623								
At1g08590	N501782								
	N440058								
At1g12460	N27942								
At1g14390	N424038								
At1g17750	N536564								
At1g34210	N503614								
At1g34420	N800007								
AT1G48480	N467014								
At1g51880	N609607								
At1g53420	N512004								
At1g51910	N831809								

Supplemental Table S1. List of T-DNA insertion lines tested for senescence phenotypes.

Primers used for Cloning	
PROMIK2-F	GAGTTTTAATATATTGATTAGG
PROMIK2-R	ACAGTTGCAGATTATCTCTCT
PROSAG12-F	ATGATTAGTTTATCATAGCTT
PROSAG12-R	TGTTTTAGGAAAGTTAAATG
GMIK2-F	ATGAACAAAACAAACCCAGAA
GMIK2-R	TTAAGAAAAGGCAGTGGAGAT
GSCOOP10-F	ATGGAAAGAAAAAAGTTTTCTT
GSCOOP10-R	CTAGGGGGCTGGAGTGCG
GSCOOP12-F	ATGAGAAATACAATTTCCTC
GSCOOP12-R	TTAATATTTTCCGTAATCTA
PMIK2-PZP211-F	CTATGACATGATTACGAATTCGAGTTTTAATATATTGATTAGG
PMIK2-PZP211-R	ACAGTTGCAGATTATCTCTCTACGGT
PMIK2-MIK2-F	GAGAGATAATCTGCAACTGTATGAACAAAACAAACCCAGAAAGA
PMIK2-MIK2-R	GGATCCCCGGGTACCGAGCTCTTAAGAAAAGGCAGTGGAGAT
PSAG12:MIK2-F	AACTTTCCTAAAACAGAGCTCATGAACAAAACAAACCCAGAA
PSAG12:MIK2-R	GGATCCCCGGGTACCGAGCTCTTAAGAAAAGGCAGTGGAGAT
PSAG12:SCOOP10-F	AACTTTCCTAAAACAGAGCTCATGGAAAGAAAAAAGTTTTCTT
PSAG12:SCOOP10-R	GGATCCCCGGGTACCGAGCTCCTAGGGGGCTGGAGTGCG
PSAG12:SCOOP12-F	AACTTTCCTAAAACAGAGCTCATGAGAAATACAATTTCCTC
PSAG12:SCOOP12-R	GGATCCCCGGGTACCGAGCTCTTAATATTTTCCGTAATCTA
35S:MIK2-GFP-F	TACCCGATCCACTAGTATGAACAAAACAAACCCAGAAAG
35S:MIK2-GFP-R	TTTACTCATAACTAGTCGAGAAAAGGCAGTGGAGATAG
**Primers used for Genotyping**	
MIK2-1-LP	TCCCCAATCTCACTTTTGTTG
MIK2-1-RP	TTTGACTTTGTTCCCAGTTGG
MIK2-2-LP	CACCGGGAATTGCTAACTCTA
MIK2-2-RP	CTGAATCTGTATGCTCCTCGA
PROSCOOP10-1-LP	TCGTACCGAATCTGCTCTTCC
PROSCOOP10-1-RP	AGCCGGAAAGAAACGTTGAAG
PROSCOOP10-2-LP	TATACTAGGGGGCTGGAGTGC
PROSCOOP10-2-RP	GAAAGAACAAGGTCATTTTGGC
08409-LB	ATATTGACCATCATACTCATTGC
LB1.3	ATTTTGCCGATTTCGGAAC
**Primers used for RT-PCR**	
MIK2-QPCR-F	TTCAAATTGGTCCGCCGTTG
MIK2-QPCR-R	GAGTTGCATCGGGAGGTGAG
SCOOP1-QPCR-F	CATGCTTCTTCTTCTTCTCAGC
SCOOP1-QPCR-R	CCTCCCAGTTATTGAGAGTTGT
SCOOP4-QPCR-F	CGTCTCCAACAAAGGAACAAAT
SCOOP4-QPCR-R	TTATCTTTAGGCGATGCAGAGT
SCOOP6-QPCR-F	CGTTATGTTGTCGTTCTGGTTT
SCOOP6-QPCR-R	ATTATTCCCCCATTCGGCATAT
SCOOP7-QPCR-F	TGTTTTGGTTCTGCTGCTTATC
SCOOP7-QPCR-R	ATTTAGGGAGTGTGTTCGTTCT

SCOOP8-QPCR-F	TTCTCAACAAGAATCACTCCCA
SCOOP8-QPCR-R	TCCACTGATTGATCCCTCAAAA
SCOOP9-QPCR-F	GTGGCTCTTCTCTGTATCTTCA
SCOOP9-QPCR-R	GTGGTTTCACATATATGCCACC
SCOOP10-QPCR-F	CGTTTCTTTCTCGTACCGAATC
SCOOP10-QPCR-R	TGGCACTGTTGGGCTTATAATA
SCOOP11-QPCR-F	TTTGCTGCTTTGTACCTTTCTC
SCOOP11-QPCR-R	TTGATGCTCCAACATCCATTTG
SCOOP12-QPCR-F	AACATTCAGTCCTACTAACCGG
SCOOP12-QPCR-R	TCAGCATCATTAAAACGTCCAC
SCOOP13-QPCR-F	GAACAGCGTGTGGATTATGATT
SCOOP13-QPCR-R	CTTTTCCTTTGCGCGATTTAGA
SCOOP14-QPCR-F	ACGCAACCTACGTAACAATTTG
SCOOP14-QPCR-R	TGATGTAGAAGGCGGTACAAAA
SCOOP15-QPCR-F	CTACAAGGTACTCGGATTCTCC
SCOOP15-QPCR-R	CTTTGGTTGAGTTAGAGTGCAC
SCOOP17-QPCR-F	AGGAGCAATGAAATTGTTGTCC
SCOOP17-QPCR-R	GGTGGCTTCTTATAAGGTTCCT
SCOOP20-QPCR-F	TATGAATCTCCAAGCCAAAGGA
SCOOP20-QPCR-R	GCACCGATCTTGAGATCTCTTA
SCOOP23-QPCR-F	AGGAAGGAAATAGGAAAAGGGG
SCOOP23-QPCR-R	ATGATCTTTTGAATGAGCTCGC
STMP3-QPCR-F	ATGTCAAGCAATGCACTTCTTC
STMP3-QPCR-R	CAATTTGCCCTCTTACTGGTTC
STMP4-QPCR-F	ATATCGCTGCATTTGTGTTACC
STMP4-QPCR-R	AGTAAATACGATAGGTGCTCCG
STMP5-QPCR-F	GTCACCAAATATTGTCGCACTT
STMP5-QPCR-R	CACGATAGGATTTCCCCAAAAC
STMP6-QPCR-F	TTTCCTCTCAGCTTAAAGTCGT
STMP6-QPCR-R	TAAGGAGATTGGTCAGAAGCAG
SAG12-QPCR -F	TCCAATTCTATTCGTCTGGTGTGT
SAG12-QPCR -R	CCACTTTCTCCCCATTTTGTTC
RBSC-QPCR-F	AGTAATGGCTTCCTCTATGC
RBSC-QPCR-R	GTGATGTCCTTGTTGGTCTTG
ACTIN-QPCR-F	TGTGCCAATCTACGAGGGTTT
ACTIN-QPCR-R	TTTCCCGCTCTGCTGTTGT

Supplemental Table S2. List of primers used in this study.

Peptide Name	Sequences (N'→C')
**SCOOP5**	IVRRSRSQRGRQY
**SCOOP6**	MISEARPSKSKKGGGR
**SCOOP7**	PNAGIRAGPSKSGQGGGR
**SCOOP8**	SNIDFEGSISGQAGGG
**SCOOP9**	GTGPSHSGHGGSS
**SCOOP10#1**	SAIGTPSSTSDHAPGSNG
**SCOOP10#2**	PNGDIFTGPSGSGHGGGR
**SCOOP11**	QMDVGASSSGQGGGR
**SCOOP12**	MGSGASGPVRSSQSSQAGGR
**SCOOP13**	RPPTAPIYLPPSKSRKGKGP
**SCOOP14**	RSMAPPIFVPPSTSHKGQGP
**SCOOP17**	KARVKRSKSKRGPQK
**SCOOP18**	KAEVGGSCSPHAHGR
**SCOOP20**	KIGASGSNSGRAPSC
**SCOOP23**	KIAVGGSDSVRAHSK
**FAM-SCOOP12**	FAM-Ahx-MGSGASGPVRSSQSSQAGGR
**FAM-SCOOP10**	FAM-Ahx-PNGDIFTGPSGSGHGGGR
**Biotin-SCOOP12**	Biotin-MGSGASGPVRSAQASQAGGR
**Biotin-SCOOP12SS/AA**	Biotin-MGSGASGPVRSSQSSQAGGR
**Biotin-SCOOP10**	Biotin-PNGDIFTGPSGSGHGGGR

Supplemental Table S3. List of peptides used in this study.
